# Oxytocin homogenizes horse group organization

**DOI:** 10.1016/j.isci.2024.110356

**Published:** 2024-06-24

**Authors:** James Brooks, Tamao Maeda, Monamie Ringhofer, Shinya Yamamoto

**Affiliations:** 1 Institute for Advanced Study, Kyoto University, Kyoto, Japan; 2 Wildlife Research Center, Kyoto University, Kyoto, Japan; 3 Research Center for Integrative Evolutionary Science, The Graduate University of Advanced Science (SOKENDAI), Hayama, Japan; 4 Department of Animal Sciences, Teikyo University of Science, Tokyo, Japan

**Keywords:** Wildlife physiology, Zoology, Evolutionary biology

## Abstract

The oxytocinergic system has been suggested to make up an important part of the endocrine basis of group cohesion. However, controlled studies in open-group settings have not been performed. We here investigated the impact of exogenous intranasal oxytocin on the group-level social organization of 5 groups of horses (*N* = 58; 12 mares and 46 geldings) through GPS tracking and social network analysis. We find oxytocin flattened social differentiation across levels. Most strikingly, oxytocin did not simply reinforce existing bonds but selectively shifted social preferences toward homogenization – individuals and pairs who otherwise rarely associated spent more time close together, while individuals and pairs with the highest baseline association instead spent more time further apart. This resulted in a more distributed structure and lower clustering coefficient at the network level. These effects reinforce and extend oxytocin’s role in collective behavior, social organization, and the evolution of group-based sociality across taxa.

## Highlights

• Exogenous oxytocin (OT) was administered to entire groups of GPS-tracked horses
• Across levels of analysis OT reduced group differentiation
• Uncommon pairs spent more time together and group-level clustering decreased
• OT’s role in group organization goes beyond reinforcement of existing dyadic bonds

## Graphical abstract

**Figure:**
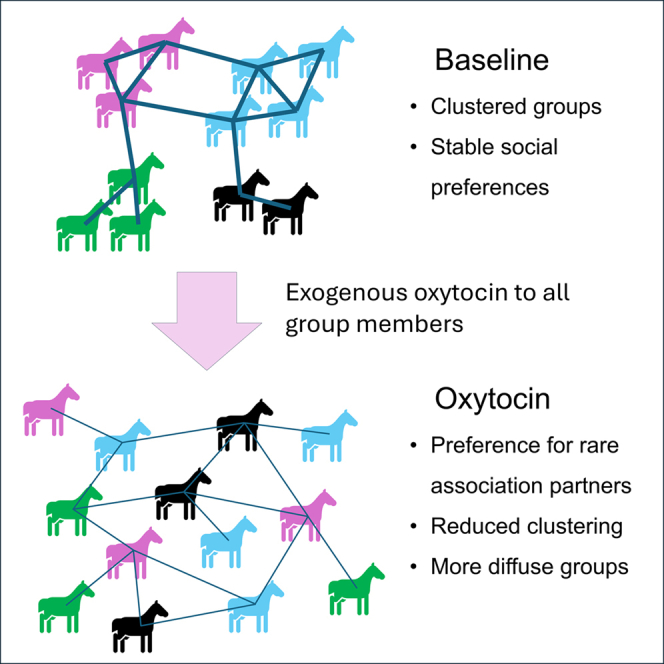

## Introduction

The hormone neuropeptide oxytocin (OT) is largely conserved across mammals and implicated in diverse social behaviors. Increasingly, studies have highlighted the peptide’s role in group-based social dynamics. These include in-group favoritism, collective action, and intergroup competition.^1^^,^^2^ One recent article reviewed evidence for a conserved “tend-and-defend” functionality of OT (and its analogs) across social vertebrates,^3^ another has dubbed it “the herding hormone”,^4^ and others have flagged it as part of the endocrine basis supporting the evolution of parochial cooperation^5^ and group-mindedness.^6^ While these studies and those they review provide evidence for a role of OT in promoting aspects of group cohesion, conformity, and cooperation, the range of contexts and species studied remains limited including, notably, a lack of studies in free-ranging settings and with herd animals. Here, we examined OT’s effect on collective behavior by analyzing the social network dynamics of groups of horses roaming in large fields after administration of either exogenous OT or saline placebo control, recorded via GPS.

OT has been studied for its influence on a range of social behavior, with pronounced research interest for its proposed effects facilitating social bonding and group cohesion. Note also the hormone has diverse roles in nonsocial domains, such as anxiolysis (anxiety reduction)^7^ and parturition.^8^ With regard to its role in social bonding, evidence from varied methodological approaches (including both measurement and administration of the hormone) has emphasized OT’s importance to the formation, maintenance, and differentiation of social bonds in the species that have received significant research attention, primarily humans,^9^^,^^10^ dogs,^11^^,^^12^ rodents,^13^^,^^14^ and non-human primates.^15^^,^^16^^,^^17^ Regarding its role in group-level cohesion, humans (*Homo sapiens*) remain the focus of most studies where psychological research, mostly in laboratory environments, has found exogenous OT promotes ethnocentrism,^18^ liking of national symbols,^19^ and coordinated outgroup attack.^20^ While more limited, studies of non-human species are consistent with a role for OT in group-based behavior and cognition. For example, recent research has found that urinary OT correlates with preparation for and involvement in intergroup encounters and collaborative hunting (contexts requiring coordination and cooperation beyond dyads) in wild chimpanzees (*Pan troglodytes*),^21^^,^^22^^,^^23^ that OT is associated with territorial behavior in captive wolves (*Canis lupus*),^24^ that exogenous OT promotes species-relevant outgroup attention in bonobos (*Pan paniscus*) and chimpanzees,^25^ and that OT is associated with aggression toward intruders in rodents.^26^^,^^27^^,^^28^ Consistent associations between OT and group-related behavior across several species, while still a narrow range of taxa, are suggestive of a potentially conserved role for OT in group-based behavior more generally. This has led some to speculate that OT may have a critical role shaping group-level social dynamics, over and above its established effects on dyadic social bonding, but empirical evidence for this hypothesis remains scant.^6^

Outside of rodents, canines, and primates, only a handful of studies have explored the role of neuropeptides on social behavior at any level. Most notably for its speculated role in group-based behavior, intravenous OT was found to promote communal activities such as guarding and pup-feeding in wild meerkats (*Suricatta suricatta*).^29^ Only one individual at a time was given OT, and therefore no group- or dyad-level effects could be directly tested, but the results highlight that OT may modulate individual-level contributions to collective behavior. In other studies, intranasal OT promoted social proximity alongside decreased vigilance in captive lions (*Panthera leo*),^30^ intravenous OT promoted mother-infant proximity in wild gray seals (*Halichoerus grypus*),^31^ and intraperitoneal injections of OT promoted huddling in captive naked mole-rats (*Heterocephalus glaber*),^32^ though none of these explored group-level effects. In birds, there are reports of a positive effect of OT homolog mesotocin on both zebra finch (*Taeniopygia guttata*) flocking behavior^33^ and pinyon jay (*Gymnorhinus cyanocephalus*) prosociality,^34^ though neither study allowed direct social interaction. Finally, isotocin treatment in a range fish species has been variously found to increase time spent associating with others (in guppies [*Poecilia reticulata*]^35^ and zebrafish [*Danio rerio*]^36^ [but see^37^^,^^38^ for contrasting results]), promote social approach (in goldfish [*Carassius auratus*]^39^), and decrease anxiety but not change social association (in *Gambusia affinis*^40^), but again the range of species and behaviors studied remain notably narrow with no attention to groups. Extreme caution must be taken before generalizing results in laboratory environments across species with different endogenous nonapeptides, socio-ecologies, and phylogenetic histories, especially considering the absence of group-level assays or consideration of existing social bond structure, but these studies are consistent with a potentially ancient role of OT (and its homologs) in social bonding and group behavior. At minimum, they stress the fundamental need for direct empirical tests in more diverse taxa, in more diverse environments, and with explicit attention toward group behavior.

We here aimed to address these research gaps through observation of group-level social organization in an open field setting. We focused on groups of domestic horses (*Equus ferus* *caballus*) with free access to large fields throughout the day, except for once- or twice-daily scheduled feedings and health checks, allowing us to balance a controlled experimental design (administration of either OT or saline placebo control following feeding) with recording of more naturistic free-moving groups of horses. While groups in this context differ from feral horse group organization in several important ways, for example being composed primarily of castrated males (see^41^), this setting permits experiments that would otherwise be impossible. Horses are an ideal model species for studies of group behavior given that they are a herding species which in natural conditions form differentiated multilevel societies^42^^,^^43^ with varied social association between members^44^ and multiyear group stability.^45^^,^^46^ They have a history of domestication,^47^ possibly shared with humans,^48^ and readily accept novel procedures without extensive training. In previous work, OT administration in horses has primarily been focused on peripheral administration in the context of veterinary studies, which support that OT is well-tolerated in horses without serious side effects.^49^^,^^50^^,^^51^ Outside a veterinary context, one thesis project investigated the behavioral effects of OT administration in horses and validated that some changes in behavior could be detected after intranasal administration.^52^ These included changes to interindividual distance, facial expressions, and handling scores,^52^ but focused on dyadic and human-directed behaviors in more constrained settings.

**Figure 1:**
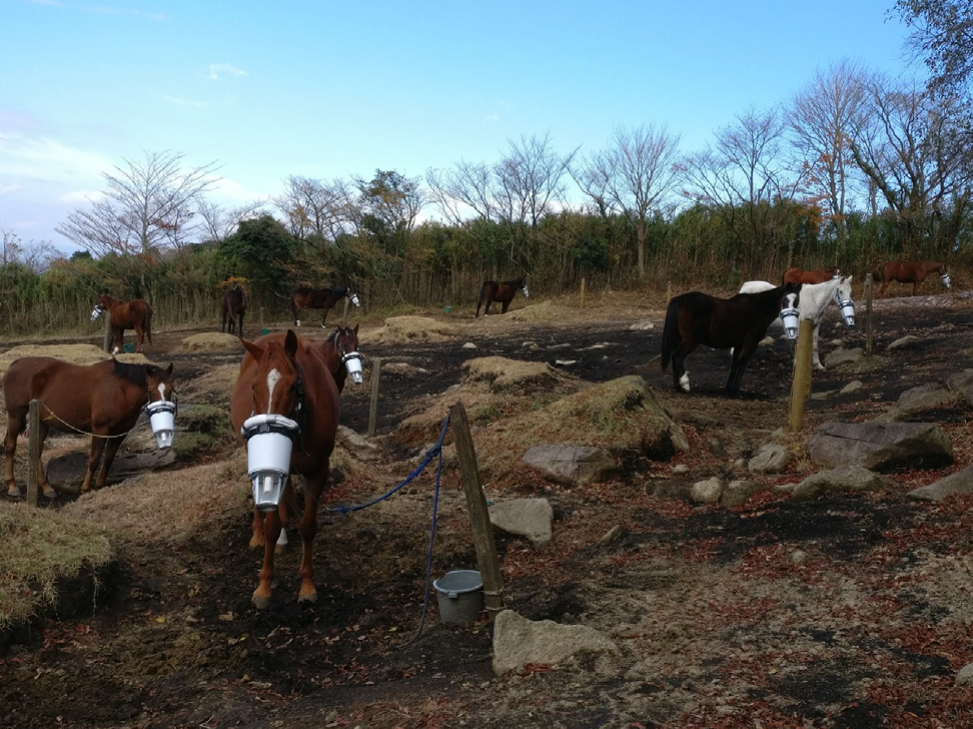
Oxytocin administration procedures to all group members simultaneously

**Figure 2:**
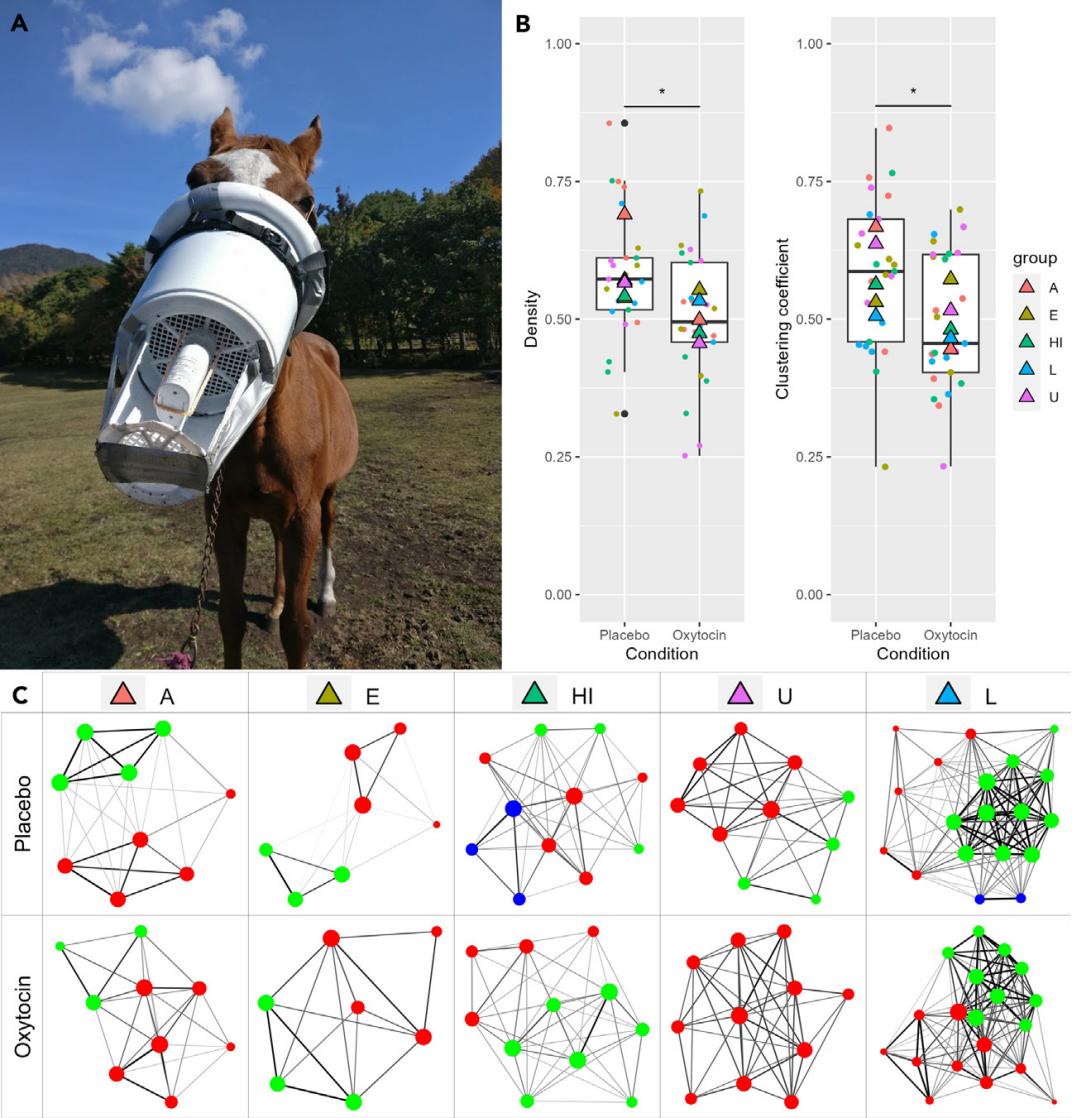
Oxytocin’s effect on group-level metrics (A) Oxytocin administration nebulizer mask design. (B) Network metrics (density and network clustering coefficient) by group and condition. Triangles represent group means, while points represent each trial. Letters refer to group names as assigned by the study site. (C) Example social networks. Each node represents a horse, line thickness represents edge weight, node size represents network centrality (strength), and color represents communities as detected by Louvain’s clustering algorithm. The bottom quartile of edges in each graph were removed to aid visualization. Note these are example networks for the purpose of illustration taken from one trial per group per condition, and not across all trials (∗ represents *p* < 0.05 in GLMM).

We measured the herd behavior of horses using GPS measurement and social network analysis, following administration of either OT or saline placebo control to entire social groups (Figures 1 and 2). We formed three general hypotheses for how OT would affect group dynamics: dyadic bonding, group-level cohesion, and the null hypothesis. While the former two are not mutually exclusive, previous literature has suggested a role for OT in group affiliation beyond its role in dyadic bonding,^3^^,^^6^ but empirical evidence remains lacking. As there are different ways the general notions of dyadic bonding and group-level cohesion could practically affect social structure, we additionally formalize, for each hypothesis, specific outcomes in network, dyad, and individual-level metrics. First, with regard to dyadic bonds, this could take the form of either a universal increase in association patterns, or a selective increase in association only in existing bonds. In the first, a universal increase in association between dyads would yield higher network density, lower diameter, and increased edge weight and strength centrality for all dyads and individuals, respectively. Alternately, dyadic bonding effects could instead accentuate existing differences by selectively promoting association among bonded pairs (if there are limitations on overall association). This would then instead result in OT making the closest dyads closer, but the more distant dyads more distant, and thereby would results in higher values for network metrics of group clustering and modularity. With regard to group-level cohesion, there are at least two distinct changes to social network structure that could be described as more cohesive (beyond simple reinforcement of all dyadic bonds). The first of these is group centralization, where groups become more centralized around key individuals. Increased association with the most central individuals would then result in less clustered and denser groups, increased association in dyads containing central individuals (but not between peripheral members), and increased differentiation of individual-level social centralities (i.e., the most central individuals would become even more central). Alternately, another form of group cohesion could instead be driven by *decreased* differentiation of group members and instead arise through group homogenization and equal status among members. This also would yield less clustered group organization (as with group centralization), but dyadic associations and individual social centralities would additionally become more uniformly distributed among group members (less differentiation between members, as opposed to centralizing around key individuals). Note that for highly centralized groups, where most associations are already with central individuals, the dyadic differentiation and group centralization yield similar predictions and may not be distinguishable, but this was not the case for the groups of horses studied here. Finally, the null hypothesis was that oxytocin, at least in this context, would exert no effect on horse social group organization. Table 1 shows the differing possible outcomes of each hypothesis.

**Table 1:** Predictions of the main hypotheses

Hypothesis	Specific outcome	Network clustering	Dyadic association indices	Network centralization
Dyadic social bond	Universal bonding	**OT = PL**	All dyads: **OT > PL**	**OT = PL**
	Dyadic differentiation	**OT > PL**	Close dyads: **OT > PL** Distant dyads: **OT < PL**	**OT < PL**
Group-level cohesion	Group centralization	**OT < PL**	With centrals: **OT > PL** Between peripherals: **OT < PL**	**OT > PL**
	Group homogenization	**OT < PL**	Close dyads: **OT < PL** Distant dyads: **OT > PL**	**OT < PL**
Null	Null	**OT = PL**	**OT = PL**	**OT = PL**

OT is OT administrated condition and PL is placebo condition.

## Results

Fifty-eight horses (12 mares and 46 geldings) in 5 social groups at Horse Trust, Kagoshima, Japan, participated in this study. Group compositions, ages, and breeds are available in supplemental information. Horses were free to graze in large fields (4–11 ha) throughout the day, except for daily feedings and health checks. OT (or saline placebo control) was administered to all horses in a group with all members. All members received the same condition within a given trial (all saline placebo or all oxytocin). Each group participated in 10 trials, half in the OT condition and half in the placebo condition. The position of each horse was then recorded at 1Hz with a GPS during free movement in home fields without human presence. To analyze how oxytocin affected the group cohesion and structure, we first built social networks for each group on each trial.^53^ Edge weights were calculated using simple ratio index (SRI), which represented the proportion of time a given dyad was in closer proximity than a set threshold.^54^ The threshold used here was the median of inter-individual distances between horses in the given group.

### Group-level metrics

From these social networks, we calculated network-level metrics including density, clustering, modularity, diameter, and sex assortativity using igraph^55^ and DirectedClustering^56^ in R.^57^ For each metric, we tested the effect of OT using a GLMM (Generalized Linear Mixed Model).^58^ Follow-up models additionally examined possible interaction with baseline values (the mean metric score across placebo condition trials) and hay provisioning (as two groups were provisioned with scattered hay throughout all trials).

We found a significant effect of OT on network-level measures of group density (β = −0.041, SE = 0.016, χ2 = 6.56, *p* = 0.014) and clustering coefficient (β = −0.044, SE = 0.018, χ2 = 6.01, *p* = 0.019) (Figure 2; Table S1). OT had no effect on diameter (χ2 = 1.29, *p* = 0.26), modularity (χ2 = 1.09, *p* = 0.30), or sex assortativity (χ2 = 0.041, *p* = 84) (Figure S3; Table S1). There was no effect of baseline values or hay provisioning on any measure (Table S1).

### Dyad-level metrics

To investigate oxytocin’s dyadic effects, we additionally analyzed network edge weights (association between each dyad) of the social networks by trial through a GLMM. Similar to the network-level metrics, follow-up models additionally examined possible interaction with baseline values (the mean metric score across placebo condition trials), sex, and hay provisioning (as two groups were provisioned with scattered hay throughout all trials).

**Figure 3:**
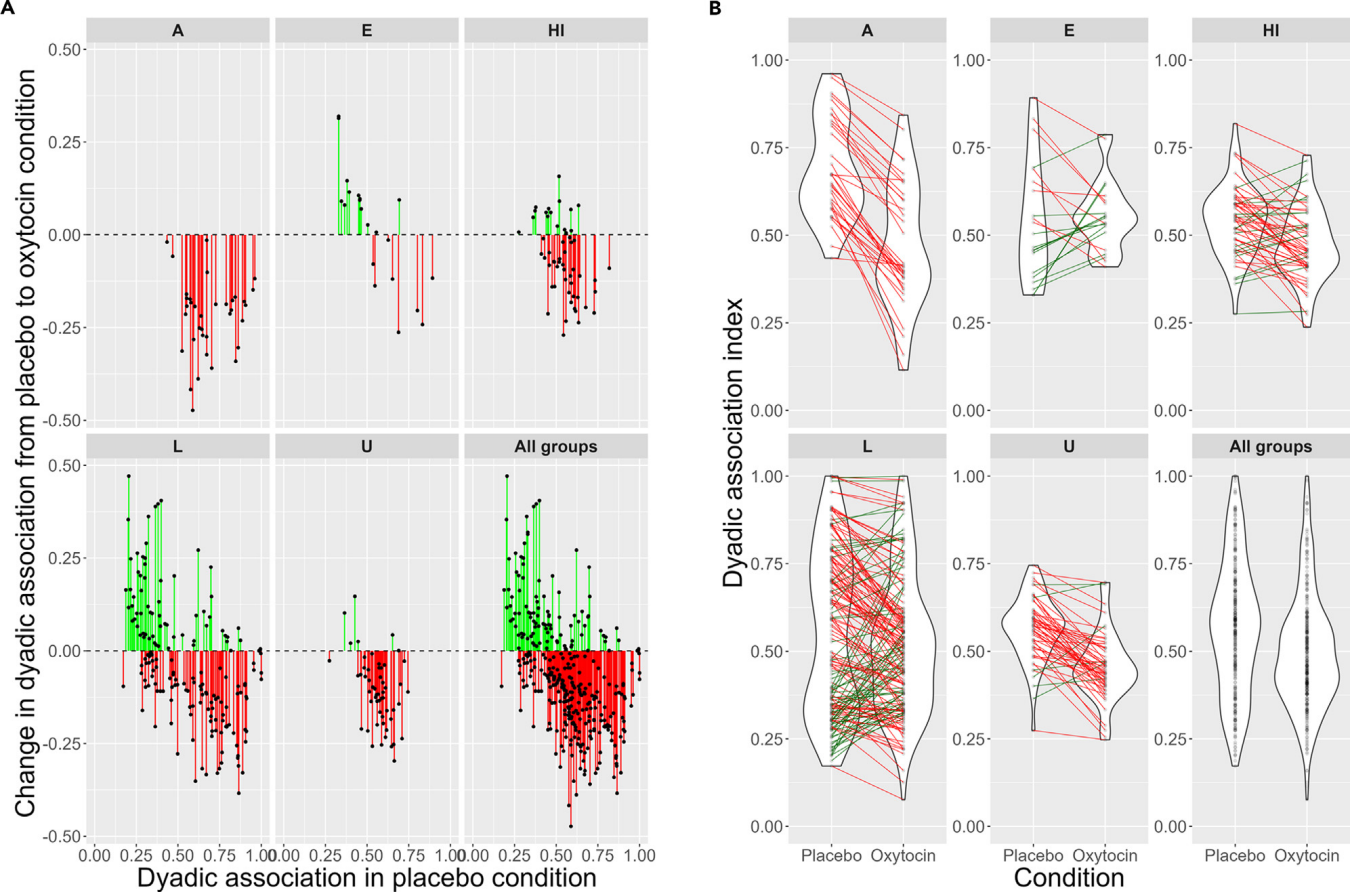
Interaction between condition and association index between all dyads across the 5 study groups Each data point represents one dyad across all trials (mean). Green indicates dyads that increased their association in the OT condition relative to placebo, and red indicates dyads that decreased their association in the OT condition relative to placebo. (A) Change in association by dyad and baseline association (placebo condition). (B) Violin plot of dyadic association index distributions by condition.

We found a significant main effect of condition (β = −0.048, SE = 0.0056, χ2 = 72.15, *p* < 0.001) and a significant interaction between baseline values and condition on dyadic proximity (β = −0.036, SE = 0.0038, χ2 = 93.46, *p* < 0.001; Figure 3; Table S2). Dyads overall were more diffuse, but dyads that were closer together during control trials drew even further apart while dyads which had been further apart in control trials drew closer together. The inter-individual distances of dyads thus became more evenly distributed. We found no main or interaction effects of hay provisioning or sex on inter-individual distances (Table S2).

### Individual-level metrics

Finally, to investigate the impact of particular individuals on oxytocin’s dyadic and network-level effects, we analyzed the network strength centrality (sum of edge weights) of each individual horse during each trial. Similar to the dyad- and network-level metrics, follow-up models additionally examined possible interaction with baseline values (the mean metric score across placebo condition trials), sex, and hay provisioning (as two groups were provisioned with scattered hay throughout all trials).

**Figure 4:**
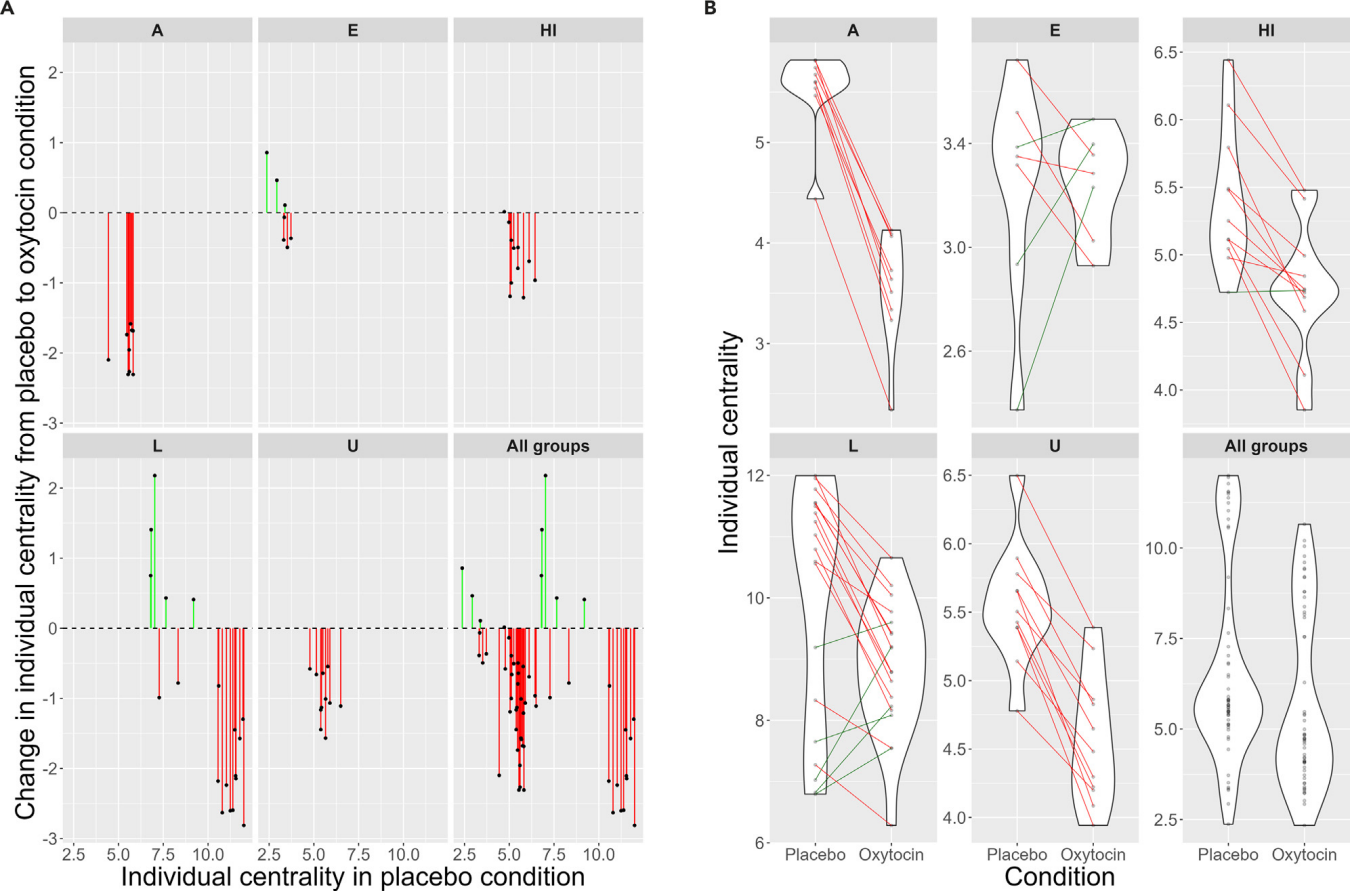
Interaction between condition and individual centrality across the 5 study groups Each data point represents one individual across all trials (mean). Green indicates individuals that increased their centrality in the OT condition relative to placebo, and red indicates individuals that decreased their centrality in the OT condition relative to placebo. (A) Change in centrality by individual and baseline centrality (placebo condition). (B) Violin plot of individual strength centrality distributions by condition.

We found a significant main effect of condition (β = −0.50, SE = 0.081, χ2 = 37.14, *p* < 0.001) and a significant interaction between baseline values and condition on individual social centrality (β = −0.15, SE = 0.030, χ2 = 24.06, *p* < 0.001; Figure 4; Table S3). Individuals overall had lower total edge weights, but the individuals who were most central during control trials became less central while individuals who were less central during control trials became more central. The centralities of individuals thus became more evenly distributed. Notably, the order of centralities between individuals remained similar, indicating the centrality pattern did not simply reverse. We found no main or interaction effects of hay provisioning or sex on inter-individual distances (Table S3).

## Discussion

On the whole, we found support across measures for group homogenization. OT decreased network clustering, within-group differentiation of dyads, and differentiation of individual social centrality, resulting in more uniform group organization. Contrary to our expectation, however, OT additionally decreased group density, through higher inter-individual distances across dyads. OT had no impact on sex assortativity or group diameter, and the presence of hay did not have a main effect or interact with any other effects. One interpretation for this suite of effects is that OT promoted whole-group cohesion while decreasing vigilance, resulting in less segregated, more uniform, but also more diffuse, social groups.

These results show that higher levels of OT can, in free-ranging settings, have group-level consequences. This partially reinforces the role of OT as “the herding hormone”^4^ and part of the endocrine basis of “group-mindedness”,^6^ but with some important caveats. OT did not selectively increase the association indices of the closest dyads, nor uniformly increase or decrease inter-individual distances, but instead weakened the differentiation of clusters, dyads and individuals. These changes may have important consequences on collective behavior. More specifically, group organization likely represents a trade-off between network structures suited to different contexts.^59^ For example, both network equitability and centralization can have positive effects on collective action,^60^^,^^61^ while higher clustering can favor cultural accumulation.^62^ Studies in some species have linked OT to collective action and group cooperation in certain contexts,^3^^,^^20^^,^^22^^,^^23^^,^^24^ and the present results suggest OT may exert some of these group effects through modulation of social group organization. Under certain conditions, OT may shift individuals’ relative disposition to associate with the group overall compared to with specific other individuals, yielding more assimilated groups that may be at an advantage for certain forms of collective action. The consequences of the network changes observed here on actual collective action more broadly cannot be determined from the current data, but the effects demonstrate a potential hormonal mechanism that can promote group-level social cohesion. If these effects can be replicated in other species and contexts, it would be powerful evidence in support of OT’s proposed role in top-down, not only bottom-up, group cooperation.^6^

The more diffuse groups (lower network density and higher average inter-individual distances) may be hypothesized to be driven in part through OT’s known anxiolytic effects (e.g., Brosnan et al.,^63^ 2015; Gamer et al.,^64^ 2010; Kirsch et al.,^7^ 2005). While closer proximity is often assumed to indicate higher social tolerance and affiliation, the socio-ecology of study species should be considered carefully. In frugivorous or carnivorous species, for example, closeness is primarily constrained by social tolerance around clumped resources to which group members want access. Some individuals therefore can gain resource access through closer distances, enabled by social tolerance and affiliation. In grazing prey species such as horses, however, resources are distributed widely and closeness instead is constrained by the risks associated with venturing too far from others. Closeness between individuals is thought to act as defense against predators,^65^^,^^66^ and as such is more likely related to social support *despite* the cost in resource access, which would instead be maximized by more diffuse feeding in the absence of threats. Through its anxiolytic effects, therefore, it is possible that OT decreased the vigilance of these horses (who face no real predatory or intergroup threats) and relaxed their dependence on close inter-individual distances. Though a different experimental approach, a similar interpretation was proposed by Brosnan et al.^63^ in finding higher inter-individual distances and lower rates of food sharing in capuchin monkeys after receiving OT compared to placebo control (in this experiment with high-value monopolizable resources and participants in individual testing chambers). In other studies, Burkhart et al.^30^ found increased social proximity in lions after receiving OT, Brooks et al.^15^ found no direct effect of OT on proximity in bonobos (though found higher rates of social grooming, which necessitates close proximity), while Mooney et al.^32^ and Smith et al.^67^ found OT promoted huddling in naked mole rats and marmosets, respectively. Together, these results argue strongly against any general role of OT on social proximity, but demonstrate its effect is species- and context-specific. In the case of horses, some support of the anxiolytic interpretation comes from Keith,^52^ which similarly found higher inter-individual distances, alongside lower heart rate, after receiving intranasal OT compared to placebo control in captive settings, though more studies are needed.

This study provides evidence that OT can alter social organization in freely associating animal social groups, making horse groups more homogeneous across levels of analysis. This reinforces the importance of OT in the evolution of group-level behavior, and tentatively supports a distinction between group and dyadic association. These results also highlight the potential for research in behavioral endocrinology outside the lab environment, particularly employing exogenous OT administration, and to understudied species. Much future work is needed, but the current findings support and extend current perspectives on OT’s role in herd behavior, collective action, and the evolution of group-mindedness.

### Limitations of the study

First, while horses were unconstrained in free-ranging contexts, they were not in fully naturalistic environments. In true feral conditions, horses form single- or multi-male harem groups, along with bachelor groups, which form a multilevel society and engage in interband aggression.^42^ In the present study, however, horses were raised mostly in captive environments, artificially formed by humans, all males were castrated, there was a strong sex bias, and there were no true outgroups. These may be serious confounds on naturalism and the generalizability of these results to horses and to group-living mammals more generally. The lack of true outgroups to test between-group assortativity and how the present effects may be generalized across more diverse social contexts are serious limitations. Similarly, there may be sex-specific effects of oxytocin that we were unable to detect owing to our study population including primarily castrated males. Interactions with other hormones in varied social contexts could not be tested here but may have significant moderating effects on the findings reported here. Additionally, oxytocin has been associated with dominance status and social hierarchy^68^ which suggests the potential for important differences between our study population and feral groups that may be targeted in future works. Second, this study made use of GPS data but did not analyze the actual social interactions or observable gross social behaviors between horses. Following similar procedures, but instead focusing on behavioral analyses with an ethological approach, will be an important future direction. Third, it cannot be determined from the present data whether the group-level effects depended on each horse receiving OT, or if OT administration only to certain group members would be sufficient to elicit the same network effects. Experiments targeting those with the highest and lowest dominance rank, social network centrality, and absolute social bond strengths can help distinguish these possibilities (of note, see Li et al.,^69^ 2022 for a study on the spread of OT-induced social changes after treatment to only central nodes in artificial human networks). Fourth, more focused physiological studies will be necessary to examine the efficiency and time-course of intranasal OT administration compared to endogenous release, and whether exogenous OT has any effect on other hormones such as cortisol in this species. Specifically, our administration procedure methods did not permit us to look at more fine-grained time-course for dyadic association throughout the period after administration, and pharmacological studies targeting change over time will be an important direction. Fifth, direct tests are needed to confirm the anxiolytic interpretation for the observed decrease in density. Future studies should compare how specific stressors (such as loud noises or predator/outgroup cues), indicators of stress (such as glucocorticoid concentrations and body posture), and anxiolytic interventions (including simple social reassurance, or compounds with known anxiolytic but not social affiliative properties) impact inter-individual distances to be compared to the OT effect reported here. Sixth, attempts should be made to measure the functional consequences of network changes detected here to animal group settings in context, for example through comparing network density to feeding efficiency and clustering to participation in collective action. Finally, similar studies in a wider range of species and settings are needed. Given oxytocin’s importance to species-typical sociality and its different proximate effects even in closely related species,^70^^,^^71^^,^^72^^,^^73^ it remains possible that OT could have a conserved role in group cohesion yet take its action through divergent specific effects. For example, while horse groups here became more cohesive through homogenization, in species that do not form herds, with more hierarchical social dynamics, or in more tense settings such as intergroup competition, oxytocin may instead promote group cohesion through other means (such as increased centralization). Alternatively, it may decrease group cohesion under some conditions and in some species. Controlled comparative work is needed, and caution should be taken until these studies can be performed. Nonetheless, this study provides important insight into OT’s role in shaping social network structure of free-moving animal groups in open-field settings.

## STAR★Methods

### Key resources table

**Table:** 

REAGENT or RESOURCE	SOURCE	IDENTIFIER
**Chemicals, Peptides, and Recombinant Proteins**		

Oxytocin	Peptide Institute Inc.	4084-v

**Software and Algorithms**		

R	R Core Team	4.2.2

### Resource availability

#### Lead contact

Further information and requests for resources should be directed to James Brooks (jamesgerardbrooks@gmail.com).

#### Materials availability

This study did not generate new unique reagents.

#### Data and code availability

• All original data is available in supplemental information.
• All original code is available in supplemental information.
• Any additional information required to reanalyze the data reported in this paper is available from the lead contact upon request.

### Experimental model and study participant details

#### Participants and study site

Fifty-eight horses (12 mares and 46 geldings, ages 1–30) in 5 social groups at Horse Trust, Kagoshima, Japan, participated in this study. Group compositions, ages, and breeds are available in Supplementary Information. Foals were included in social network analysis but were not given OT or placebo treatment. Horses were free to graze in large fields (4–11 ha) throughout the day, except for daily feedings and health checks once (for four groups) or twice (for one group) per day. During this time, horses were tied at designated locations and received a bucket of ground hay. Food provided during daily feedings was constant across days for each horse, but varied between horses according to size, age, and health condition. All experimental protocols were conducted during the morning feedings for each group. Outside of this feeding time, horses roamed in their fields without human interaction. Each social group has access to a separate field and thus there was no chance of inter-group encounters. In the colder months, where less grass is available for grazing, horses are provisioned with scattered hay in their fields (distributed during feedings). Data collection of 3 groups (L, A, and E) occurred prior to hay being provisioned, while in 2 groups (U and HI), hay was provisioned. Within each group, each trial was consistent (either no trial or every trial had scattered hay) and amounts of provisioned hay per group were fixed. In all groups, water was available from a large water tub near the morning feeding area. Group membership was constant across data collection periods for all social groups, except for one horse being moved prior to the final trial in both L and U groups (due to a hoof injury and stomach-ache, respectively).

Horse Trust is a non-profit organization providing naturalistic lives and care to retired horses. As such, participants had a variety of life-history backgrounds, including as racing and riding club horses. In one group (L), there were two foals (1 year of age) who roamed freely with the group and were thus included in GPS data collection and social network analysis, but did not receive OT treatment. All other participants were adults (>4 years of age).

### Method details

#### Ethics statement

This research was approved by all relevant ethical committees and conformed to local and international regulatory standards on animal care. Horses were never food or water deprived at any time. Ethical approval number from the Wildlife Research Center ethical committee was WRC-2021-007A.

Dose for this study was chosen based on several factors. While there is variability in the strategies chosen (e.g., Brosnan et al.^63^ adjusting dosage for weight, or Burkhart et al;^30^ using a fixed dose on the lower end of the human spectrum), we based our own consideration on two sources of information, as well as some practical consideration. First, Keith^52^ was the first to address exogenous oxytocin in horse social behavior, and using a similar dose (there, 150 IU) did not report any adverse effects. Secondly, a line of veterinary studies targeting OT in ungulates (e.g.,^74^) similarly used body weight-adjusted dose finding no welfare concerns and that it was well-tolerated by participants. Direct discussion with veterinary researchers working in this area also led us to consider this an appropriate dose. Finally, on the more practical side, while we nebulized a fixed dose of 158IU to each horse in a systematic, consistent, and controlled manner, we do not expect 100% of this reached their nasal passages, and less still into their brain and bloodstream. Besides general challenges in non-restraint administration paradigms about mist escaping out the edges of the mask (e.g.,^73^^,^^75^), horses in particular are expected to have a lower proportion of the nebulized dose reach their brain and bloodstream. Specifically, compared to lions or primate, horses nasal passages and the path to the olfactory epithelial cells for transport to the brain is significantly longer. A large amount of the mist is therefore expected to condensate before reaching the olfactory epithelial cells, meaning the dose that reached the brain of each horse was likely significantly less than the dose which was nebulized.

#### Nebulizer mask design

Nebulizer masks were custom made for this study. The basic design consisted of a bucket, waterproof foam padding lining the inside, a nose bridge (in 3 sizes suited for the horse’s snout size), and additional light foam padding around the bucket rim for safety (Figures 1 and S1). Adjustable straps were then attached, which could go over the horse’s head behind the ears, and tightened until secure, and featured a quick release mechanism. An additional strap went under the horse’s chin and around both ends of the main strap to keep it taut and away from horses’ eyes. On the bottom of the bucket, a hole for the nebulizer was drilled, and a nebulizer attachment glued into place. A smaller bucket with one side removed was then attached to the bottom to protect the nebulizer (while keeping it accessible to human experimenters). Finally, a rope was secured to the bottom of the bucket which could tightly go over the nebulizer when attached, keeping it secure while horses moved their heads freely.

#### OT administration procedures

OT (or saline placebo control) was administered to horses immediately upon finishing their breakfast. A portable nebulizer (Omron NE-U100) was used to create a mist of OT, which was inserted into a custom-made nebulizer mask designed to keep the mist around the horses’ noses, where they could passively breathe the nebulized mist (Figures 1, S1, and S2). The solutions were volumetrically dosed, and nebulizers were thus inserted into the custom mask until all the solution had been nebulized (approximately 10 min per horse). Administration procedures started as soon as horses finished their breakfast, and the entire administration process was completed in under 1 h (from attaching the first nebulizer to removing the last nebulizer) for all horses in a social group on each trial.

We used a fixed dose of 158IU of OT dissolved in 2.5mL of saline. We chose this dose considering the scaling of intranasal studies of OT typical of macaque and human studies (see^76^^,^^77^) by species-average body weight, and following previous behavioral studies of OT administration in horses.^52^

#### GPS data collection

GPS recorders (TranSystem Inc. GL-770) were attached to each horse during their feedings and the administration procedures. GPSs recorded each horse’s position at 1Hz frequency. GPSs was replaced each trial to download the recorded data and recharge devices. For most horses (all but one), GPSs were attached to their halters using cable ties. The remaining horse did not like wearing halters and would remove them if attached, and thus his GPS was instead attached to his front left leg using a custom leg band. On 6 occasions, a GPS was either not recovered (likely fell off during free roaming) or failed to record positional data.

#### Data collection strategy

Each group participated in 10 trials (5 OT and 5 placebo) across up to 3 weeks. Trials were conducted at a maximum of once per day, where administration procedures were completed immediately following their morning feeding and health check. Each member of a social group received the same treatment condition (either all members received OT or all received saline placebo control). Trial orders were pseudo-randomized, such that groups never received the same treatment more than twice consecutively or in four of five consecutive trials. Trial-by-trial information is available in the Supplementary Information.

One hour of data per group was analyzed per trial, starting from 30 min after the last group member completed OT administration procedures. All data in this period thus was at least 30 min after the end of OT administration for each horse, and at most 2.5 h after the beginning of OT administration for each horse, following previous studies of exogenous OT administration of other species.^76^^,^^77^^,^^78^^,^^79^

### Quantification and statistical analysis

For all models, significance was calculated using chi-squared likelihood ratio test with the drop1 function^80^ which uses full—null model comparison for hypothesis testing and an alpha value of 0.05.

#### Group-level analyses

To analyze how oxytocin affected the group cohesion and structure, we first built social networks for each group on each trial.^53^ Edge weights were calculated using simple ratio index (SRI), which represented the proportion of time a given dyad was in closer proximity than a set threshold.^54^ The threshold used here was the median of inter-individual distances between horses in the given group. In order to remove the possible bias from missing data, we first took the median distance across all recorded timepoints for each dyad, and then took the median of these dyadic scores per group (thereby ensuring that all individuals and dyads are weighted equally even with unequal missing datapoints). From these social networks, we then calculated network-level metrics including density, clustering, modularity, diameter, and sex assortativity. All the network metrics were calculated using R package “igraph”.^81^ As supplemental analysis, we also used a threshold of median distance across all dyads in all groups and performed the same analyses (results were consistent with either choice of threshold, full details are available in Table S4).

Density is the proportion of realized edge weights to the sum of possible edges.^82^ Clustering coefficient is the tendency of the neighboring nodes of any given node to also be connected to one another, i.e., to form a clique (fully connected set of nodes). We used Barrat’s method for calculating clustering coefficient.^83^ Modularity measures the likelihood of edges to be connected within clusters compared to between clusters, indicating how well networks are divided into sub-groups.^84^ We identified communities using Louvain’s clustering algorithm.^85^^,^^86^ Diameter is the largest value of the shortest paths between all the pairs in a network.^53^ Sex assortativity measures how likely individuals are to be closer to the same sex individuals, where assortativity was calculated using Newman’s method.^87^

We then ran GLMMs on these network metrics to see how network characteristics differ between control and oxytocin condition. We used a fixed effect of condition and random effects of date and group. We additionally ran subsequent models to check the impact of provisioned hay and baseline network metrics (the average metric score in control condition days), by adding an interaction with condition as a fixed effect. If the interaction between hay provisioning and condition was not significant, we ran the model again removing the interaction to examine the possible main effects of hay provisioning on group-level metrics.

#### Dyad-level analyses

To investigate oxytocin’s effect on dyadic association, we additionally ran a GLMM on the network edge weights (association between each dyad in each trial). This model again with fixed effect of condition and a random effect of day, as well as random effects of dyad ID, ID1, and ID2, where dyad ID was a unique variable for each dyad, and ID1 and ID2 were the two individuals making up the dyad, randomly designated ID1 or ID2 for each data point. This was done following previous multi-membership model approaches to capture random effect structure across dyads themselves composed of overlapping individuals (e.g., see^88^^,^^89^^,^^90^). Similar to the group-level analyses, we additionally ran subsequent models to explore the impact of hay provisioning and baseline dyadic closeness, as well as sex. As supplemental analyses, we also ran these models using mean absolute distances for each dyad per trial as the response variable, as well as edge weights using a between-group threshold (results were similar across measures, full details for both models available in Tables S5 and S6).

#### Individual-level analyses

To investigate the impact of particular individuals on oxytocin’s dyadic and network-level effects, we analyzed the network strength centrality (sum of edge weights) of each individual horse during each trial. We ran a GLMM on this measure of network centrality, again with fixed effect of condition and random effect of day, as well as a random effect of individual ID nested in group. Similar to the group-level analyses, we additionally ran subsequent models to explore the impact of hay provisioning and baseline centrality, as well as sex. As supplemental analyses, we also ran these models using eigenvector centrality as the response variable, as well as strength centrality using a between-group threshold (results were similar across all models, full details available in Tables S7, S8, and S9).

## Supplemental information

Document S1. Figures S1–S4 and Tables S1–S9

Data S1. All data and code described in this manuscript, related to STAR Methods Contains all original data, processing code, formatted data, analysis, and figure code.
