# Supplementary material for: Oxytocin homogenizes horse group organization
Source: iScience. 2024 Jun 24;27(7):110356. doi: 10.1016/j.isci.2024.110356 (PMC11277748; doi:10.1016/j.isci.2024.110356)
Supplement: Document S1. Figures S1–S4 and Tables S1–S9 [file mmc1.pdf]

iScience, Volume *27*

Supplemental information

Oxytocin homogenizes horse group organization

James Brooks, Tamao Maeda, Monamie Ringhofer, and Shinya Yamamoto

Supplementary Information

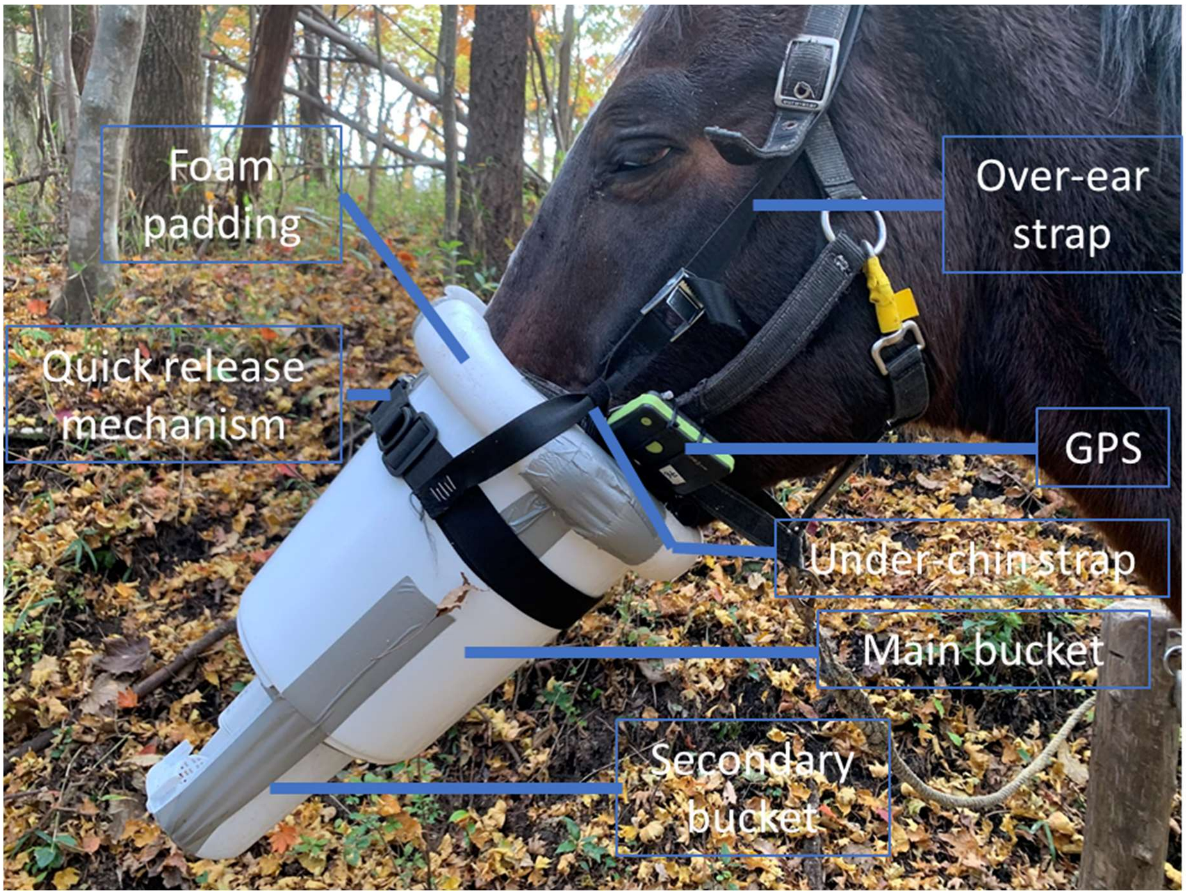

Figure S1. Side view of nebulizer mask design, related to STAR Methods.

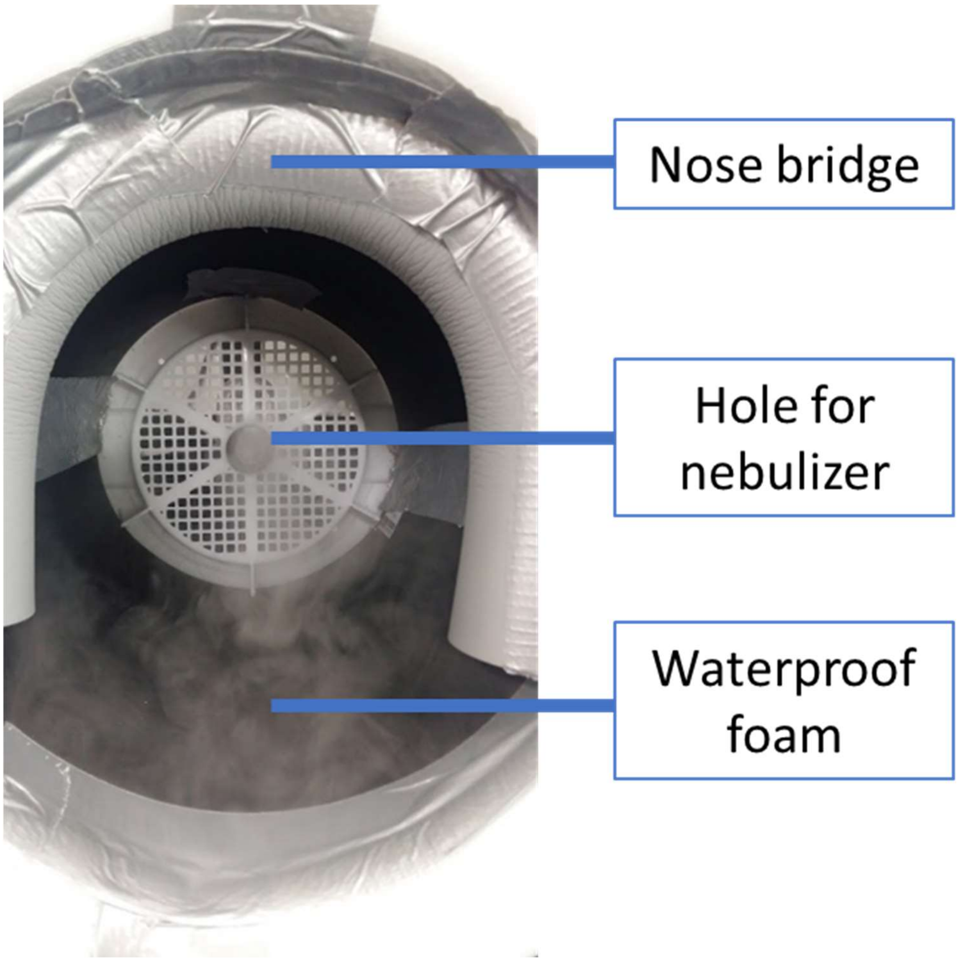

Figure S2. Front view of nebulizer mask design, related to STAR Methods.

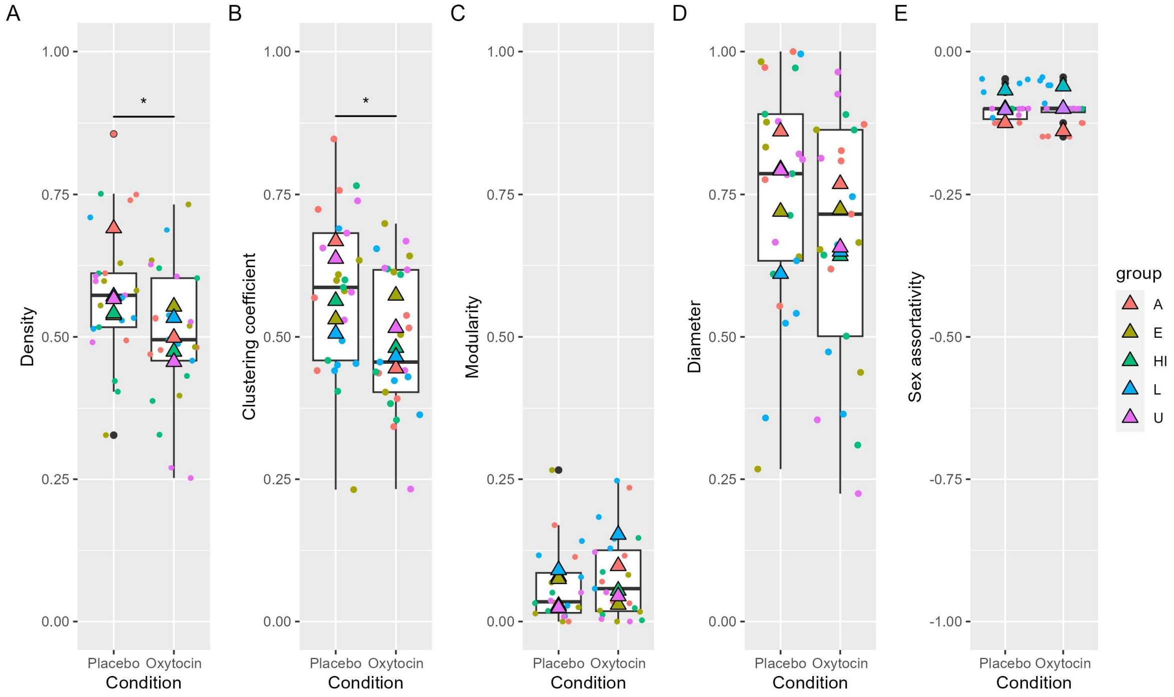

Figure S3. All network metrics by group and condition, related to Figure 2. A) Network density B) Network clustering coefficient C) Modularity (Louvain's method) D) Diameter E) Sex assortativity. Triangles represent group means, while points represent each trial.

Table S1. Full network metrics results, related to Figure 2. Bold with asterisk indicates a significant effect ($p < 0.05$) and superscript plus sign indicates a nonsignificant trend ($p < 0.1$). Test statistics are calculated using the drop1 function, which compares a model with the specified fixed effects (full) to a null model which removes only the fixed effect or interaction being tested.

Network metric	Predictor	Estimate	SE	χ2	*p*
**Density**	**Condition**	**-0.041**	**0.016**	**6.56**	**0.014***
	Condition × baseline	-0.52	-0.29	3.17	0.082^{+}
	Condition × hay provisioning	-0.0022	0.016	0.018	0.89
	Hay provisioning	-0.028	0.018	2.33	0.14
**Clustering coefficient**	**Condition**	**-0.044**	**0.018**	**6.01**	**0.019***
	Condition × baseline	-0.59	0.29	4.08	0.050^{+}
	Condition × hay provisioning	-0.0041	0.019	0.49	0.83
	Hay provisioning	0.0073	0.021	0.12	0.74
Diameter	Condition	-0.035	0.031	1.29	0.26
	Condition × baseline	-0.35	0.35	1.00	0.32
	Condition × hay provisioning	-0.031	0.031	1.03	0.32
	Hay provisioning	-0.0010	0.036	<0.001	0.98
Modularity	Condition	0.0095	0.0091	1.09	0.30
	Condition × baseline	-0.0087	0.32	<0.001	0.98
	Condition × hay provisioning	0.0023	0.0093	0.059	0.81
	Hay provisioning	-0.025	0.014	3.46	0.16
Sex assortativity	Condition	-<0.001	0.0019	0.041	0.84
	Condition × baseline	0.14	0.093	2.35	0.13
	Condition × hay provisioning	<0.001	0.0019	0.21	0.65
	Hay provisioning	<0.001	<0.001	0.0073	0.94

Table S2: Full dyadic association results, related to Figure 3. Bold with asterisk indicates a significant effect ($p < 0.05$) and superscript plus sign indicates a nonsignificant trend ($p < 0.1$). Test statistics are calculated using the drop1 function, which compares a model with the specified fixed effects (full) to a null model which removes only the fixed effect or interaction being tested.

Model	Term	Estimate	SE	χ2	*p*
**Model 1**	**Condition**	**-0.048**	**0.0056**	**72.15**	**<0.001*** **
**Model 2**	**Condition × baseline value**	**-0.036**	**0.0038**	**93.46**	**<0.001*** **
Model 3	Condition × hay provisioning	0.018	0.012	2.60	0.11
	Hay provisioning	-0.043	0.039	1.19	0.28
Model 4	Condition × baseline value × hay provisioning	0.0012	0.013	0.0082	0.93
Model 5	Condition × sex	MF: 0.0075<br>MM: 0.0086	MF: 0.021<br>MM: 0.019	1.10	0.33
	Sex	MF: 0.0014<br>MM: 0.017	MF: 0.044<br>MM: 0.043	0.45	0.64

Table S3. Full individual centrality results, related to Figure 4. Bold with asterisk indicates a significant effect ($p < 0.05$) and superscript plus sign indicates a nonsignificant trend ($p < 0.1$). Test statistics are calculated using the drop1 function, which compares a model with the specified fixed effects (full) to a null model which removes only the fixed effect or interaction being tested.

Model	Term	Estimate	SE	χ2	*p*
**Model 1**	**Condition**	**-0.50**	**0.081**	**37.14**	**<0.001*** **
**Model 2**	**Condition × baseline value**	**-0.15**	**0.030**	**24.06**	**<0.001*** **
Model 3	Condition × hay provisioning	0.099	0.083	1.41	0.24
	Hay provisioning	-0.32	1.23	0.070	0.81
Model 4	Condition × baseline value × hay provisioning	0.040	0.11	0.14	0.71
Model 5	Condition × sex	0.026	0.16	0.026	0.87
	Sex	0.34	0.30	1.24	0.27

Supplementary analyses

Table S4. Across-group median distances as threshold for network metrics, related to STAR Methods. Bold with asterisk indicates a significant effect (p < 0.05) and superscript plus sign indicates a nonsignificant trend (p < 0.1). Test statistics are calculated using the drop1 function, which compares a model with the specified fixed effects (full) to a null model which removes only the fixed effect or interaction being tested.

Network metric	Term	Estimate	SE	χ2	*p*
**Density**	**Condition**	**-0.036**	**0.016**	**4.87**	**0.033***
	Condition × baseline value	0.024	0.098	0.061	0.81
	Condition × hay provisioning	-0.012	0.017	0.57	0.45
	Hay provisioning	0.083	0.089	0.85	0.42
**Clustering coefficient**	**Condition**	**-0.039**	**0.017**	**5.01**	**0.031***
	Condition × baseline value	0.0089	0.089	0.010	0.92
	Condition × hay provisioning	-0.017	0.018	0.93	0.34
	Hay provisioning	0.11	0.10	1.26	0.34
Diameter	Condition	-0.023	0.023	0.98	0.33
	Condition × baseline value	-0.0096	0.083	0.013	0.91
	Condition × hay provisioning	-0.022	0.023	0.92	0.34
	Hay provisioning	0.17	0.13	1.72	0.28
Modularity	Condition	0.015	0.0078	3.75	0.059^{+}
	Condition × baseline value	0.18	0.11	2.66	0.11
	Condition × hay provisioning	-0.0036	0.0079	0.20	0.65
	Hay provisioning	-0.053	0.038	1.95	0.26
Sex assortativity	Condition	<0.001	0.0023	<0.001	0.99
	Condition × baseline value	0.057	0.097	0.34	0.57
	Condition × hay provisioning	<0.001	0.0023	0.040	0.84
	Hay provisioning	-<0.001	0.018	0.065	0.82

Table S5. Across-group median distances as threshold for dyad edge weights, related to STAR Methods. Bold with asterisk indicates a significant effect (p < 0.05) and superscript plus sign indicates a nonsignificant trend (p < 0.1). Test statistics are calculated using the drop1 function, which compares a model with the specified fixed effects (full) to a null model which removes only the fixed effect or interaction being tested.

Model	Term	Estimate	SE	χ2	*p*
**Model 1**	**Condition**	**-0.041**	**0.0050**	**66.85**	**<0.001*** **
**Model 2**	**Condition × baseline value**	**-0.014**	**0.0040**	**12.55**	**<0.001*** **
Model 3	Condition × hay provisioning	-0.0041	0.010	0.15	0.69
	Hay provisioning	0.18	0.20	0.83	0.43
**Model 4**	**Condition × baseline value × hay provisioning**	**0.040**	**0.020**	**4.02**	**0.045**
Model 5	Condition × sex	MF: 0.0058<br>MM: 0.0060	MF: 0.019<br>MM: 0.017	0.062	0.94
	Sex	MF: -0.0015<br>MM: 0.015	FM: 0.046<br>MM: 0.044	0.73	0.48

Table S6. Raw mean distance for dyad association, related to STAR Methods. Bold with asterisk indicates a significant effect ($p < 0.05$) and superscript plus sign indicates a nonsignificant trend ($p < 0.1$). Test statistics are calculated using the drop1 function, which compares a model with the specified fixed effects (full) to a null model which removes only the fixed effect or interaction being tested.

Model	Term	Estimate	SE	χ2	*p*
**Model 1**	**Condition**	**2.91**	**0.33**	**77.74**	**<0.001*** **
**Model 2**	**Condition × baseline value**	**-0.57**	**0.24**	**5.47**	**0.019***
**Model 3**	**Condition × hay provisioning**	**-2.67**	**0.69**	**15.08**	**<0.001*** **
	Hay provisioning	-8.89	11.38	0.61	0.49
Model 4	Condition × baseline value × hay provisioning	0.40	0.90	0.20	0.66
Model 5	Condition × sex	MF: 1.10<br>MM: 1.82	MF: 1.23<br>MM: 1.12	1.74	0.18
	Sex	MF: 0.51<br>MM: -1.52	MF: 2.61<br>MM: 2.48	1.72	0.19

Table S7. Within-group median distances as threshold for individual centrality (eigenvector), related to STAR Methods. Bold with asterisk indicates a significant effect ($p < 0.05$) and superscript plus sign indicates a nonsignificant trend ($p < 0.1$). Test statistics are calculated using the drop1 function, which compares a model with the specified fixed effects (full) to a null model which removes only the fixed effect or interaction being tested.

Model	Term	Estimate	SE	χ2	*p*
Model 1	Condition	-0.013	0.0071	3.55	0.060^{+}
**Model 2**	**Condition × baseline value**	**-0.32**	**0.45**	**50.11**	**<0.001*** **
Model 3	Condition × hay provisioning	-0.0043	0.0071	0.36	0.55
	Hay provisioning	-<0.001	0.021	0.0020	0.97
Model 4	Condition × baseline value × hay provisioning	0.077	0.070	1.20	0.27
Model 5	Condition × sex	0.0043	0.016	0.076	0.78
	Sex	0.026	0.033	0.66	0.42

Table S8. Across-group median distances as threshold for individual centrality (strength), related to STAR Methods. Bold with asterisk indicates a significant effect ($p < 0.05$) and superscript plus sign indicates a nonsignificant trend ($p < 0.1$). Test statistics are calculated using the drop1 function, which compares a model with the specified fixed effects (full) to a null model which removes only the fixed effect or interaction being tested.

Model	Term	Estimate	SE	χ2	*p*
**Model 1**	**Condition**	**-0.44**	**0.066**	**44.65**	**<0.001*** **
Model 2	Condition × baseline value	-0.064	0.035	3.29	0.070^{+}
Model 3	Condition × hay provisioning	0.0051	0.068	0.0056	0.94
	Hay provisioning	1.09	0.63	2.99	0.17
**Model 4**	**Condition × baseline value × hay provisioning**	**0.23**	**0.088**	**6.63**	**0.010***
Model 5	Condition × sex	0.067	0.13	0.26	0.61
	Sex	0.40	0.27	2.23	0.14

Table S9. Across-group median distances as threshold for individual centrality (eigenvector), related to STAR Methods. Bold with asterisk indicates a significant effect ($p < 0.05$) and superscript plus sign indicates a nonsignificant trend ($p < 0.1$). Test statistics are calculated using the drop1 function, which compares a model with the specified fixed effects (full) to a null model which removes only the fixed effect or interaction being tested.

Model	Term	Estimate	SE	χ2	*p*
Model 1	Condition	-0.016	0.0080	3.82	0.052^{+}
**Model 2**	**Condition × baseline value**	**-0.12**	**0.041**	**8.62**	**0.0035****
Model 3	Condition × hay provisioning	-0.0035	0.0081	0.19	0.66
	Hay provisioning	0.057	0.048	1.39	0.32
Model 4	Condition × baseline value × hay provisioning	-0.0032	0.10	0.0010	0.98
Model 5	Condition × sex	0.0076	0.018	0.18	0.67
	Sex	0.060	0.043	1.97	0.17

A

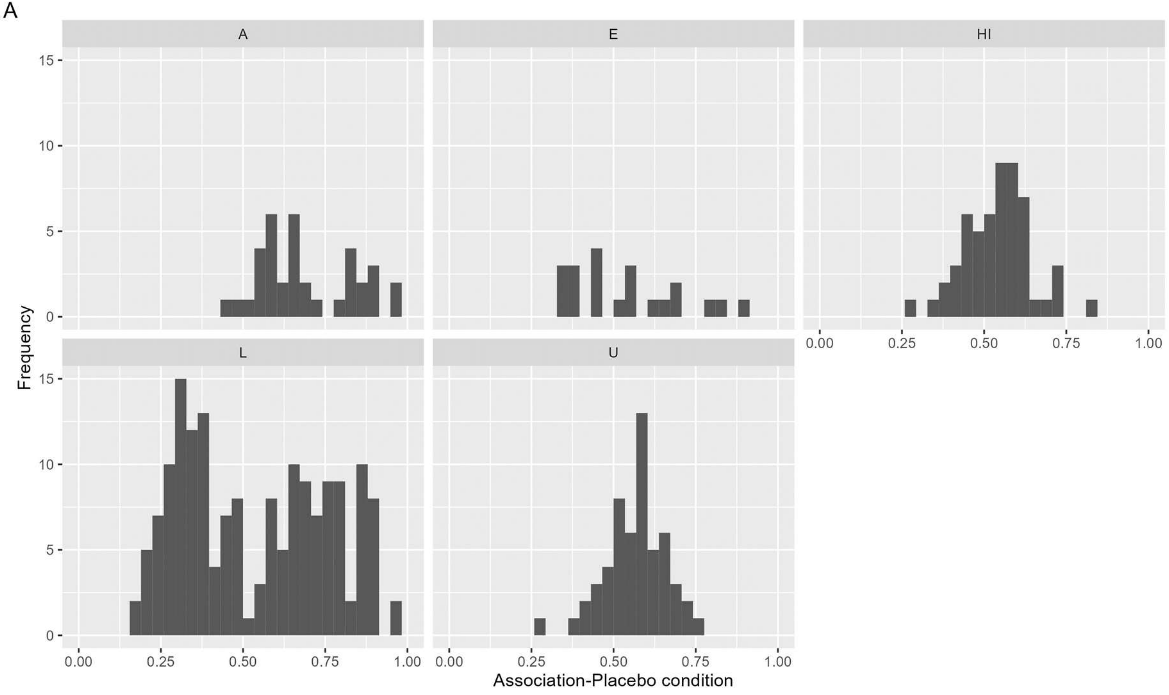

B

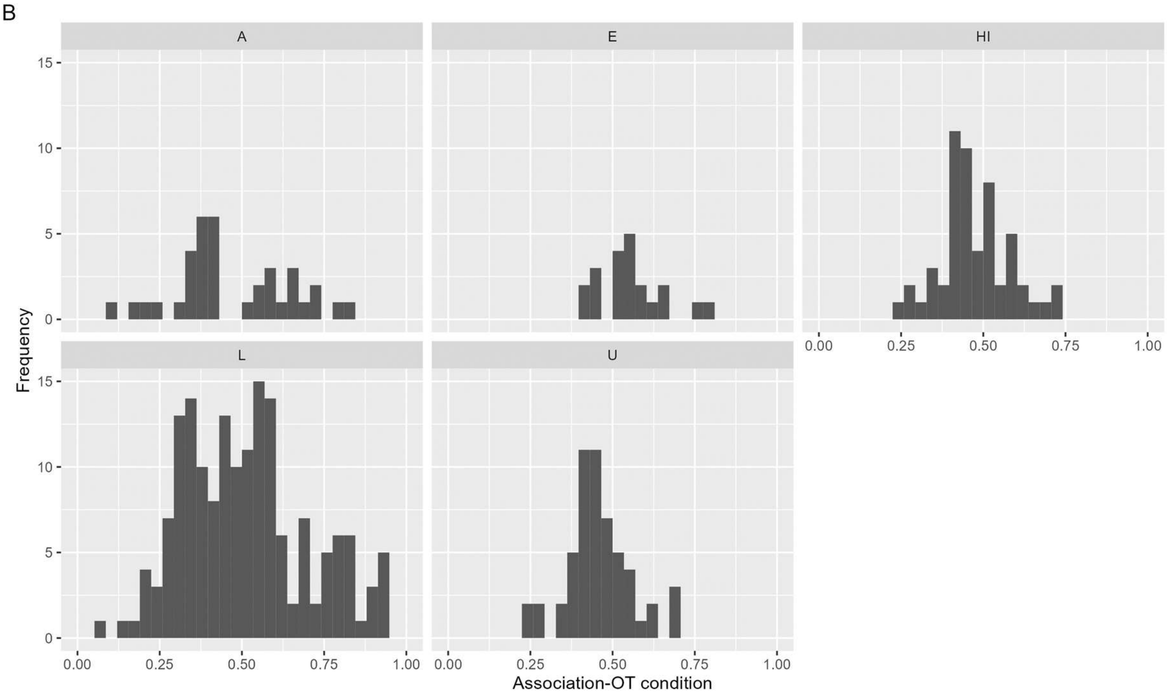

Figure S4. Histogram of dyadic association indices by group and condition, related to STAR Methods. A) Placebo condition B) Oxytocin condition.
