# Supplementary figures and images for: Oxytocin homogenizes horse group organization
Source: iScience. 2024 Jun 24;27(7):110356. doi: 10.1016/j.isci.2024.110356 (PMC11277748; doi:10.1016/j.isci.2024.110356)

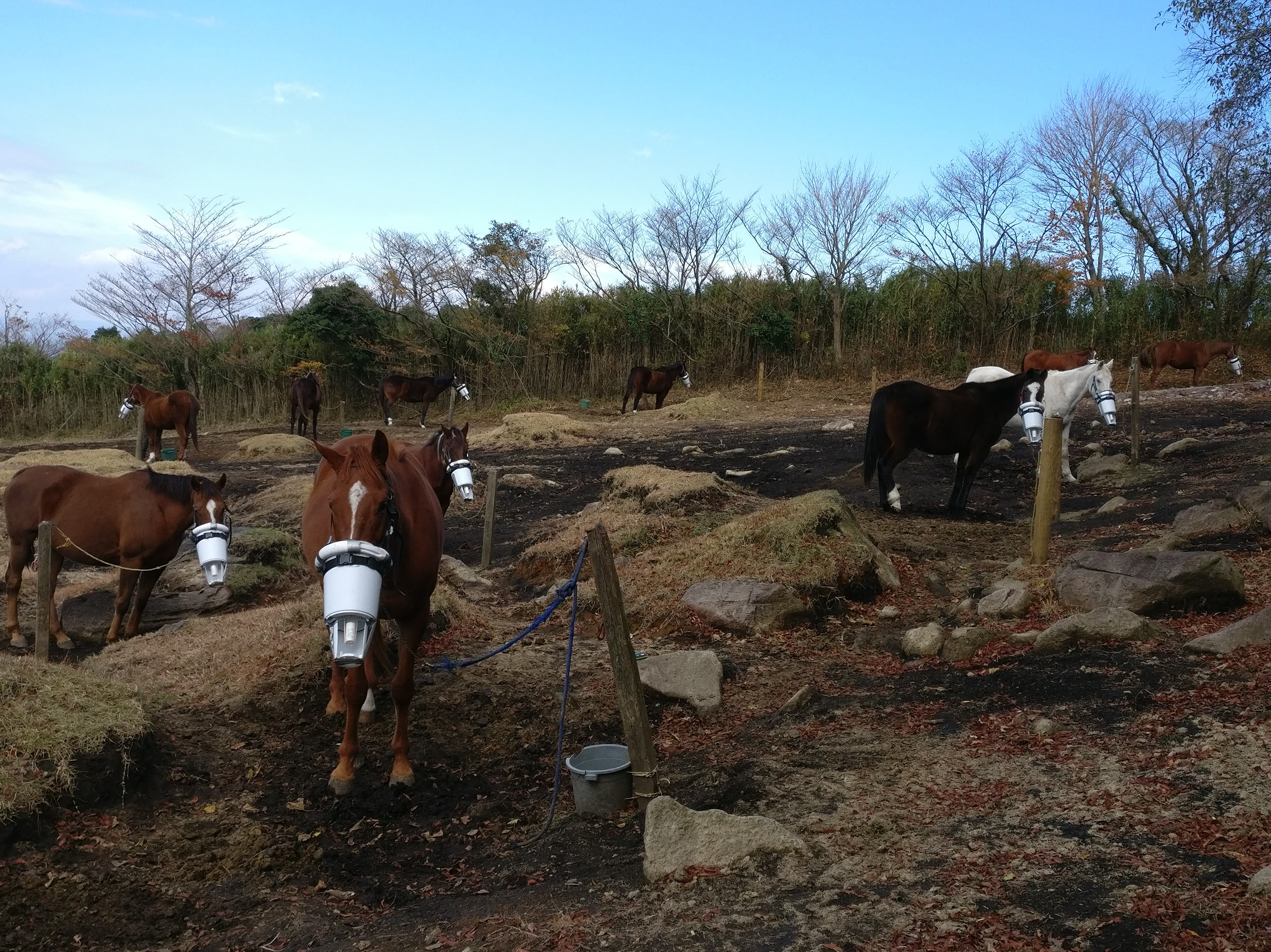

Supplement: Data S1. All data and code described in this manuscript, related to STAR Methods — Contains all original data, processing code, formatted data, analysis, and figure code. [file mmc2.zip › figures/Fig1.tiff]

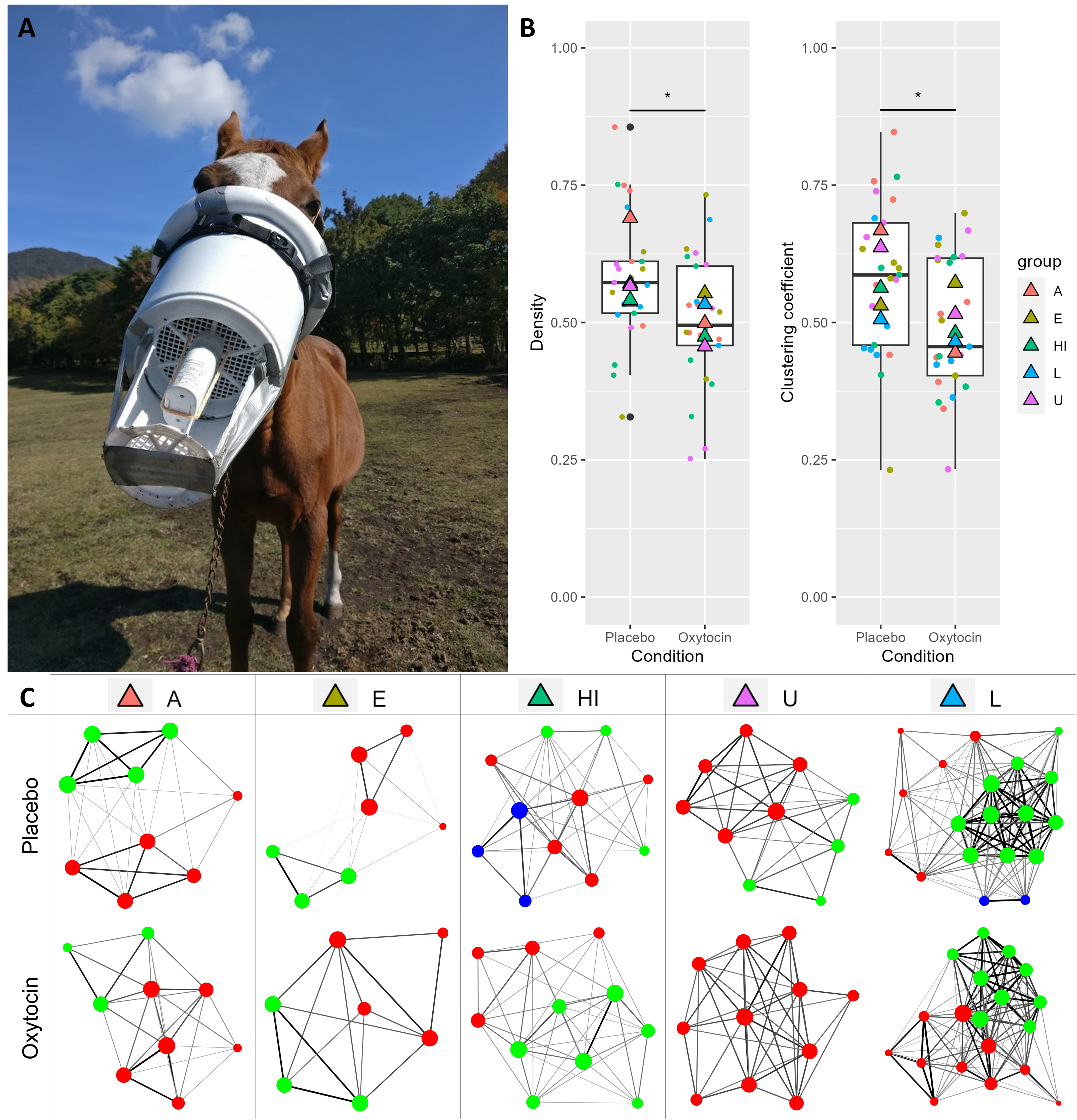

Supplement: Data S1. All data and code described in this manuscript, related to STAR Methods — Contains all original data, processing code, formatted data, analysis, and figure code. [file mmc2.zip › figures/Fig2.tiff]

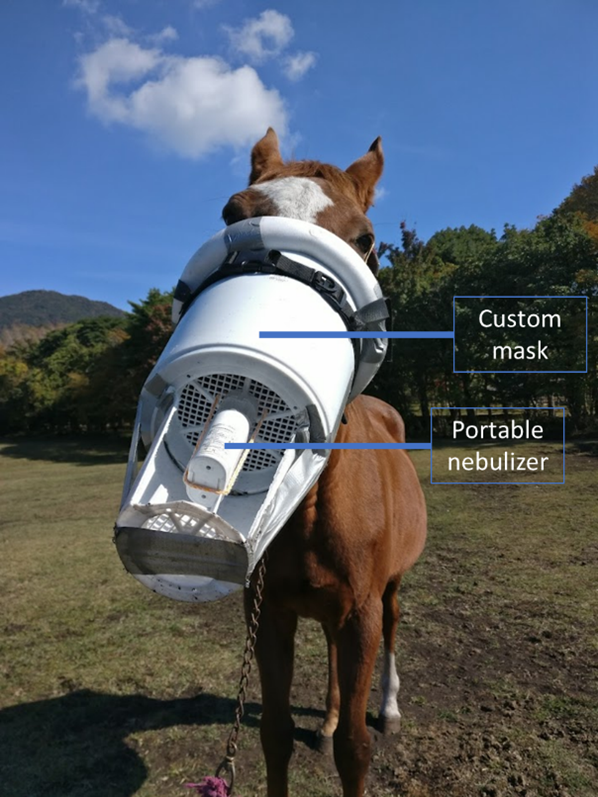

Supplement: Data S1. All data and code described in this manuscript, related to STAR Methods — Contains all original data, processing code, formatted data, analysis, and figure code. [file mmc2.zip › figures/Fig2a.png]

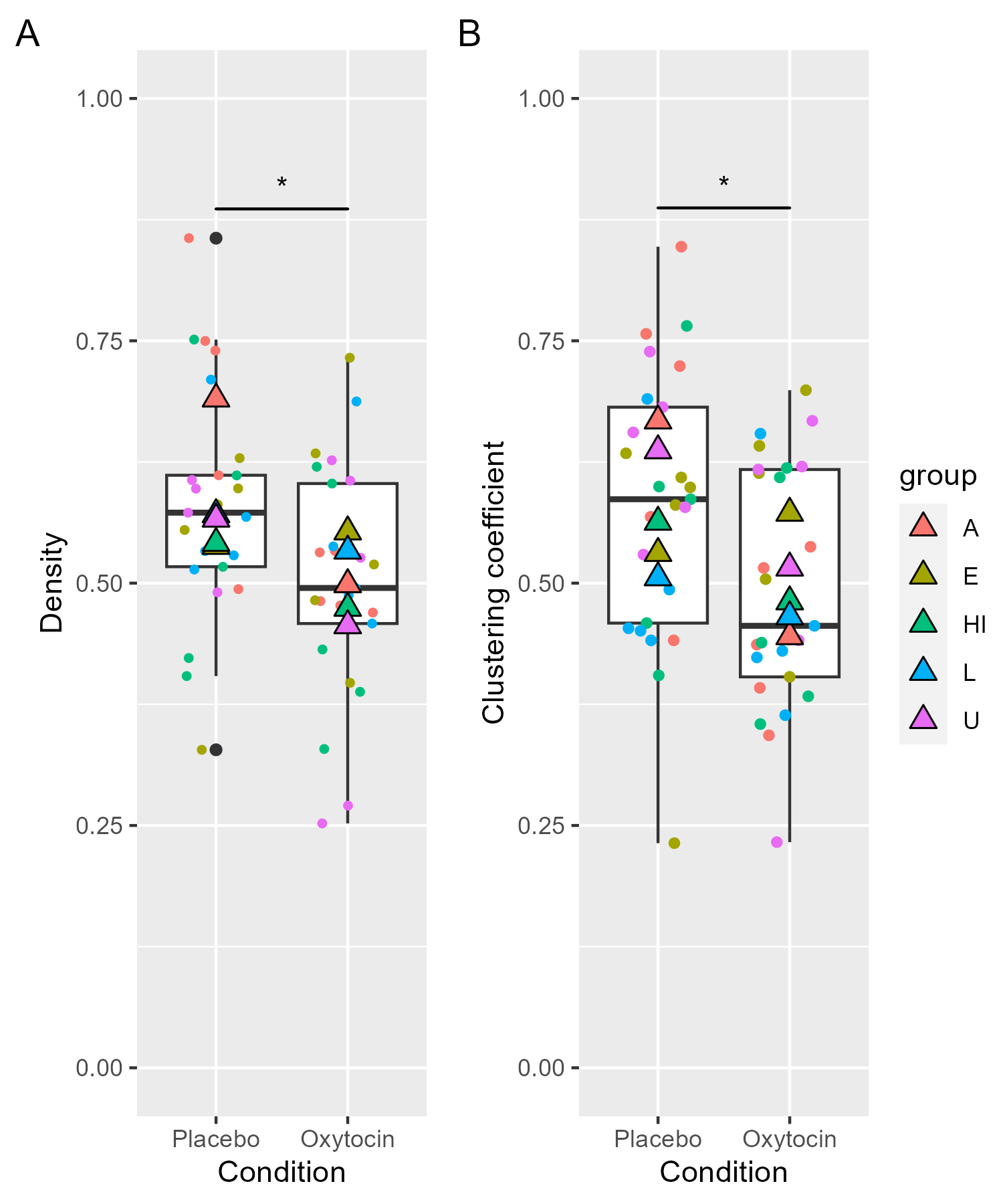

Supplement: Data S1. All data and code described in this manuscript, related to STAR Methods — Contains all original data, processing code, formatted data, analysis, and figure code. [file mmc2.zip › figures/Fig2b.png]

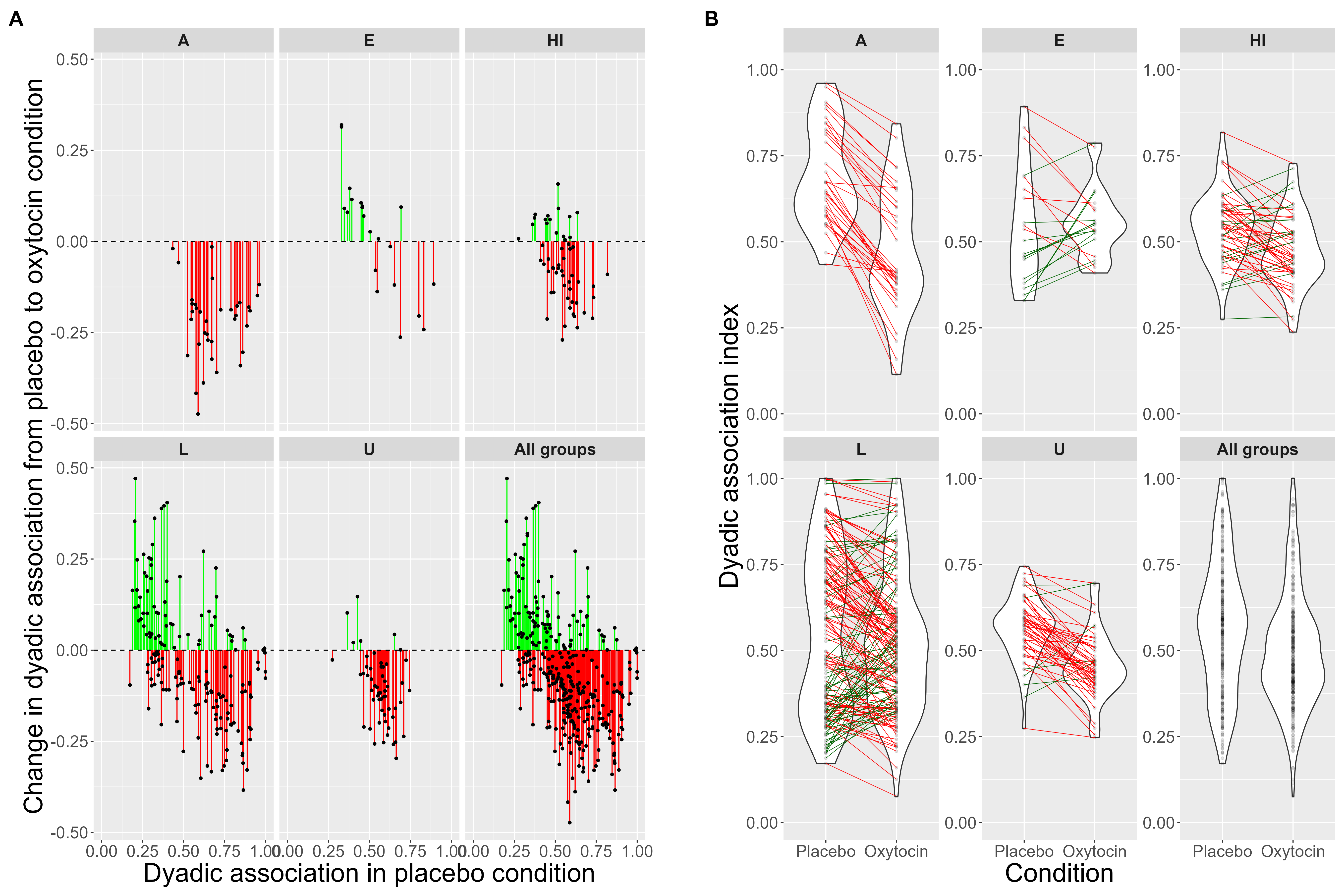

Supplement: Data S1. All data and code described in this manuscript, related to STAR Methods — Contains all original data, processing code, formatted data, analysis, and figure code. [file mmc2.zip › figures/Fig3.tiff]

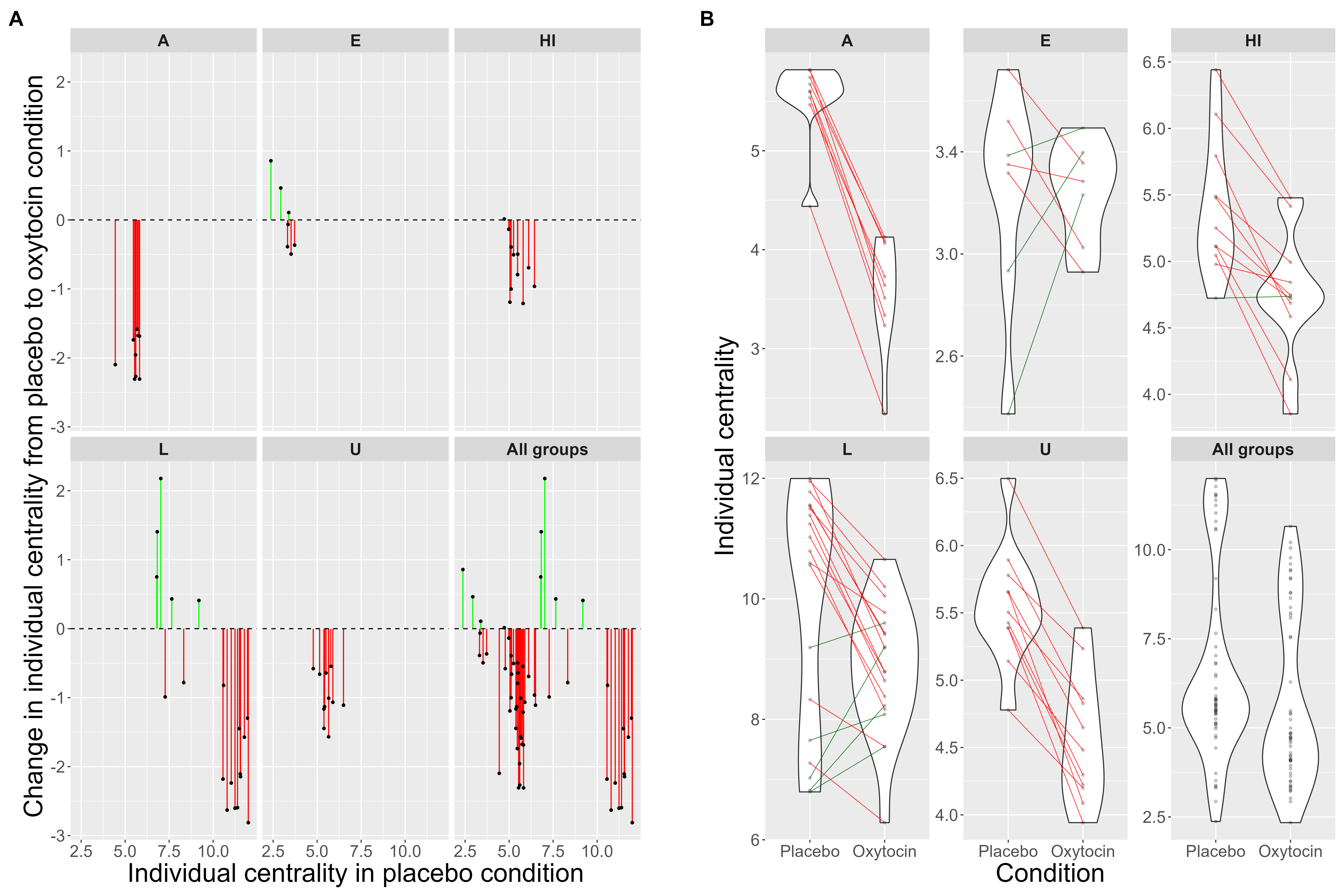

Supplement: Data S1. All data and code described in this manuscript, related to STAR Methods — Contains all original data, processing code, formatted data, analysis, and figure code. [file mmc2.zip › figures/Fig4.tiff]

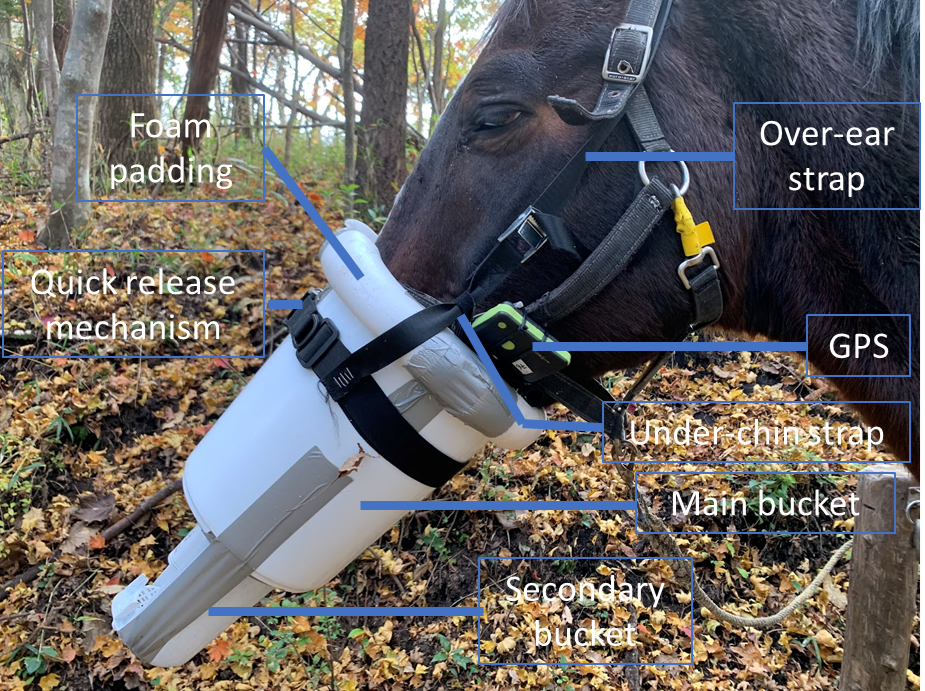

Supplement: Data S1. All data and code described in this manuscript, related to STAR Methods — Contains all original data, processing code, formatted data, analysis, and figure code. [file mmc2.zip › figures/FigS1.png]

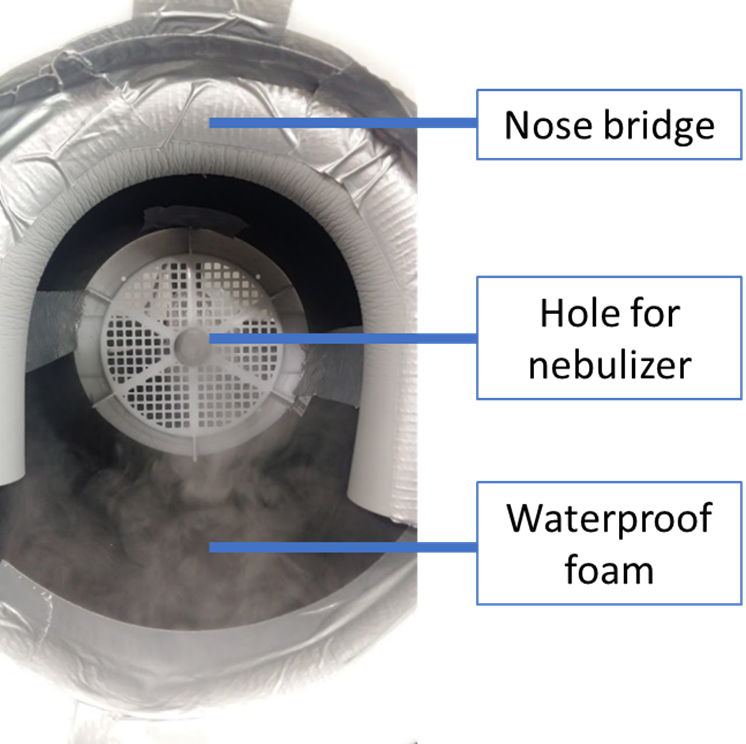

Supplement: Data S1. All data and code described in this manuscript, related to STAR Methods — Contains all original data, processing code, formatted data, analysis, and figure code. [file mmc2.zip › figures/FigS2.png]

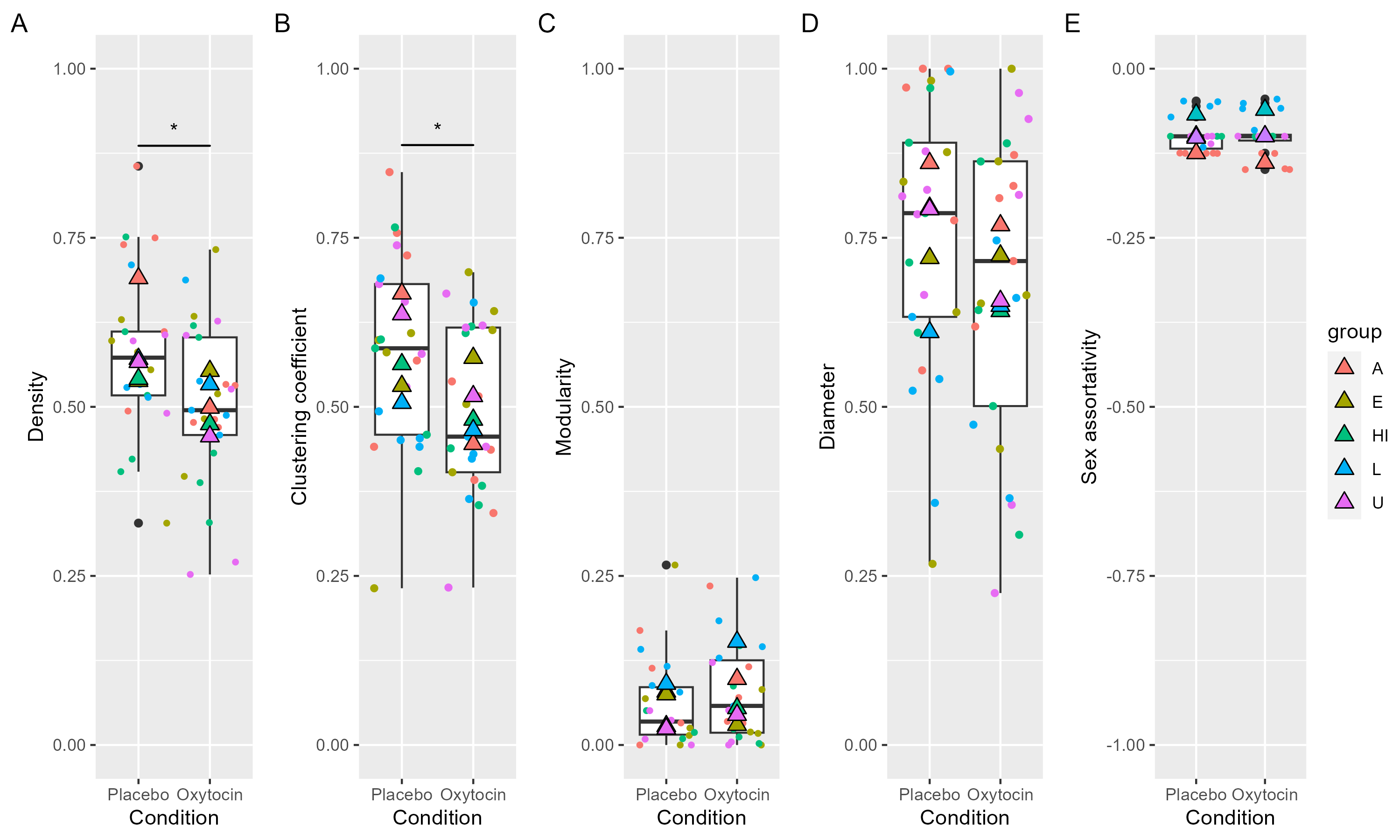

Supplement: Data S1. All data and code described in this manuscript, related to STAR Methods — Contains all original data, processing code, formatted data, analysis, and figure code. [file mmc2.zip › figures/FigS3.png]

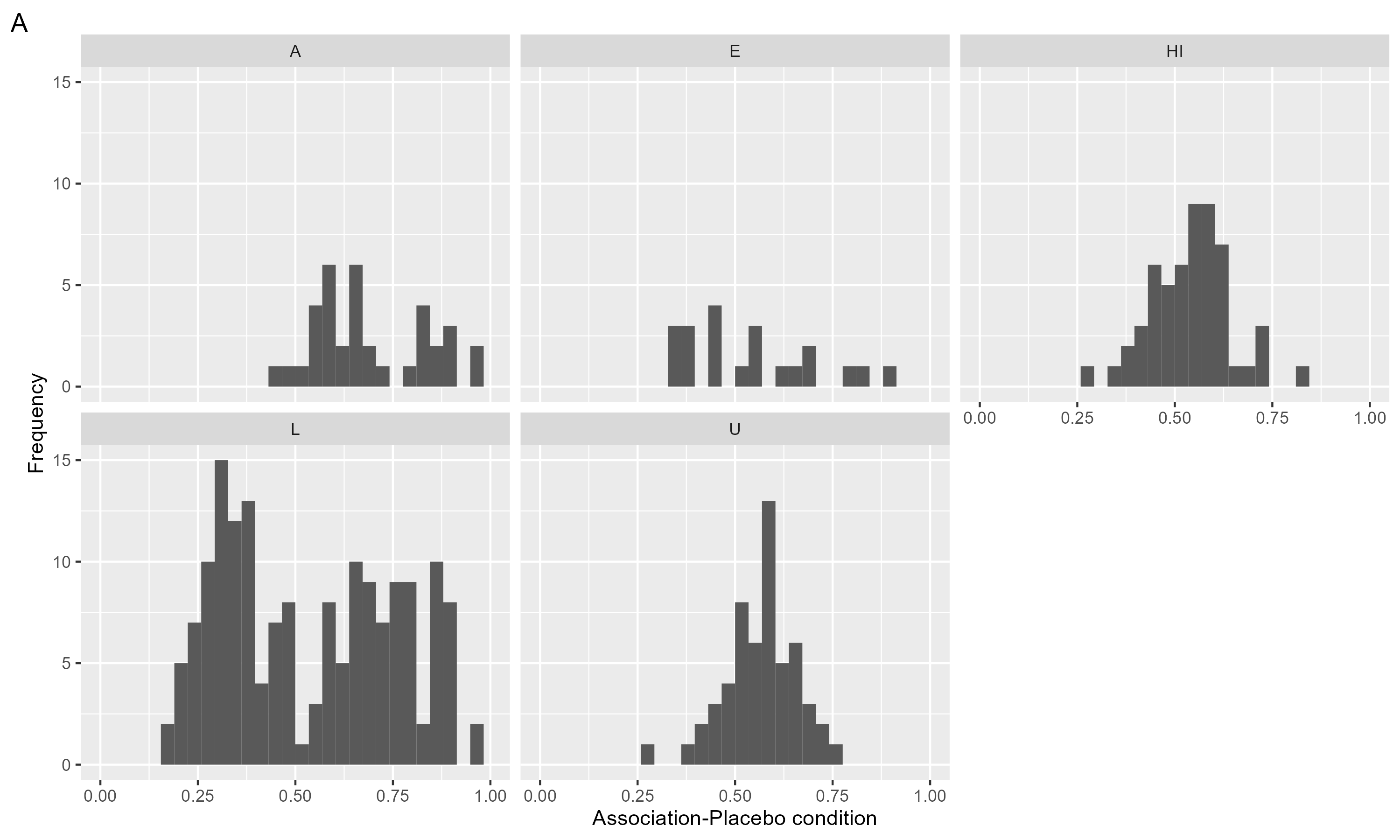

Supplement: Data S1. All data and code described in this manuscript, related to STAR Methods — Contains all original data, processing code, formatted data, analysis, and figure code. [file mmc2.zip › figures/FigS4a.png]

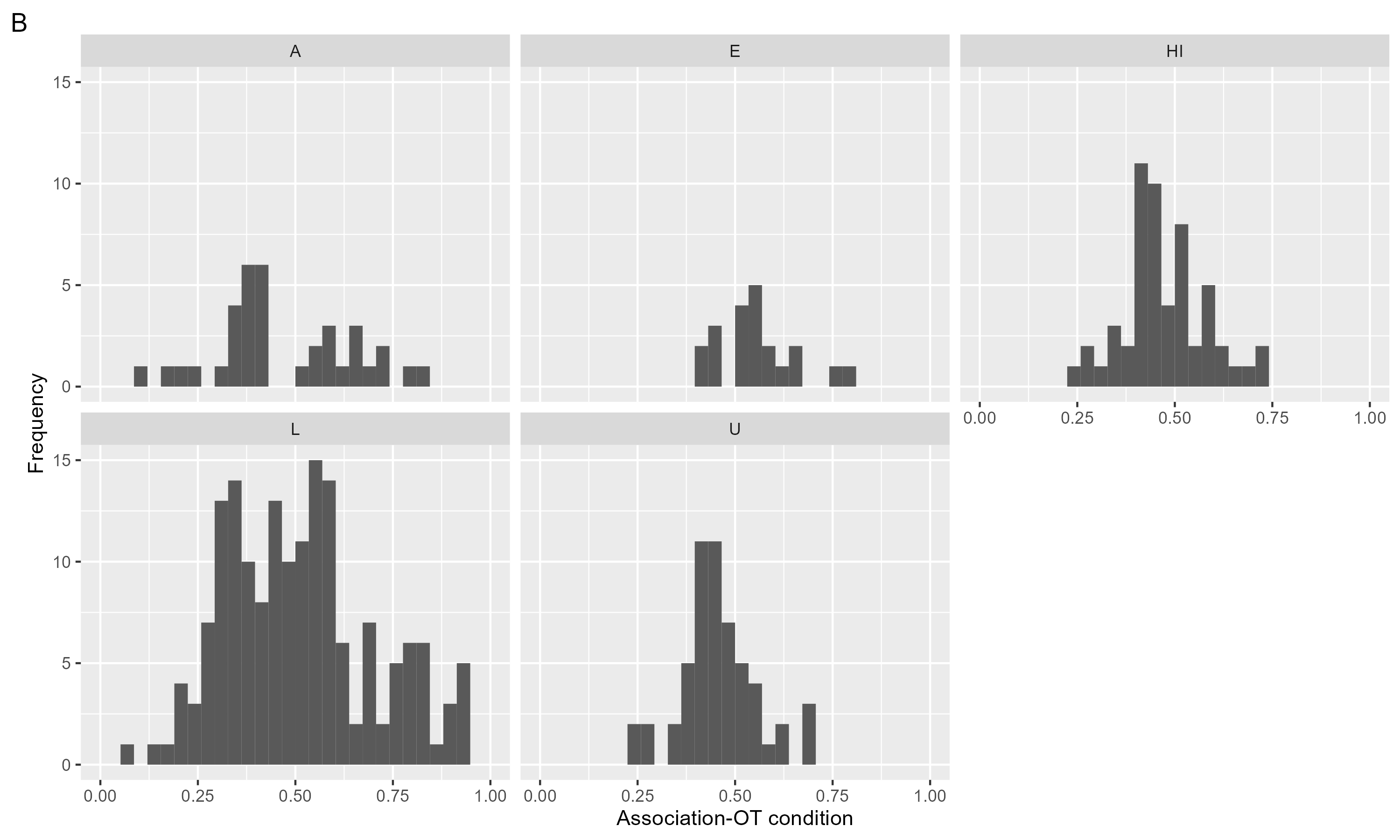

Supplement: Data S1. All data and code described in this manuscript, related to STAR Methods — Contains all original data, processing code, formatted data, analysis, and figure code. [file mmc2.zip › figures/FigS4b.png]

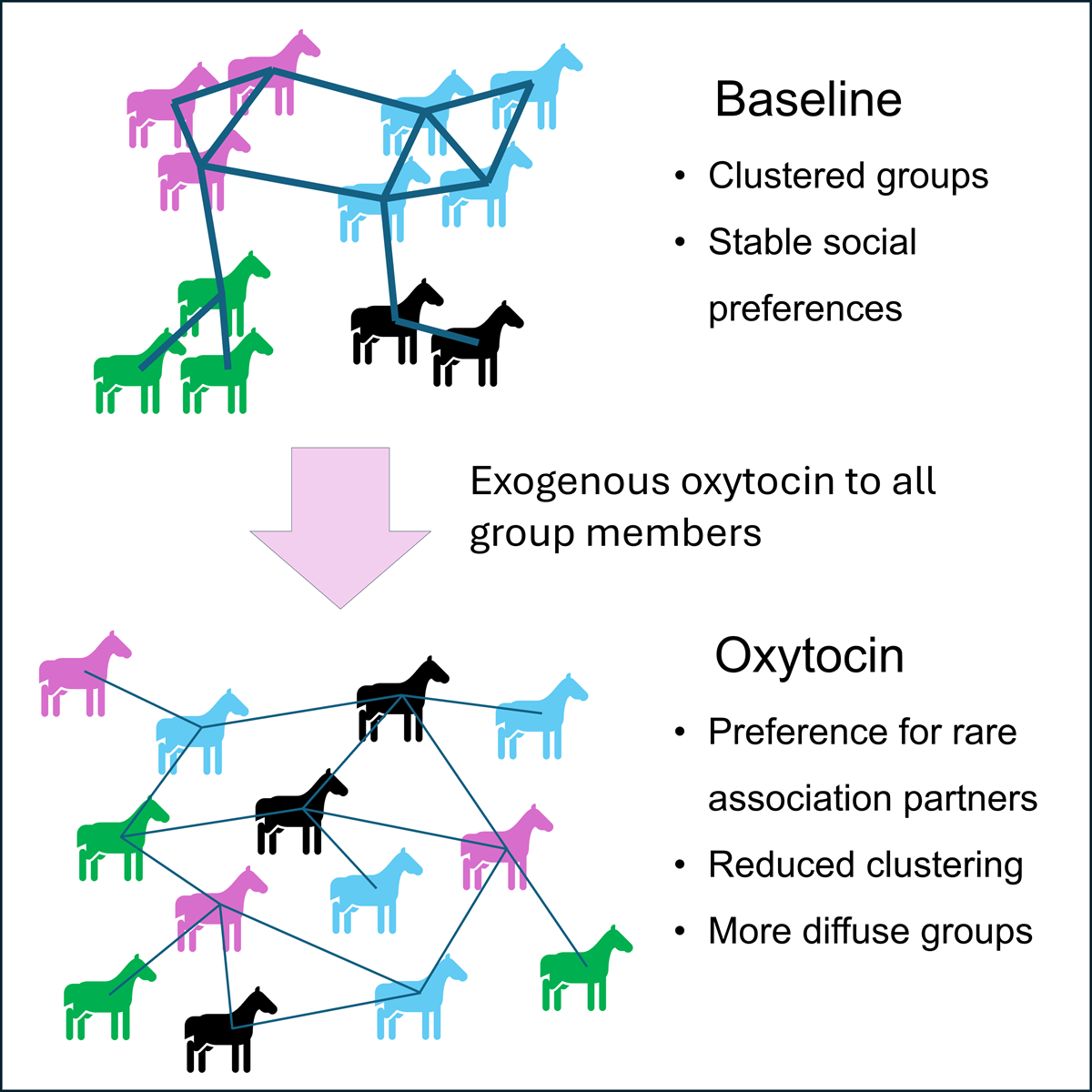

Supplement: Data S1. All data and code described in this manuscript, related to STAR Methods — Contains all original data, processing code, formatted data, analysis, and figure code. [file mmc2.zip › figures/GraphicalAbstract.tif]
